# Genome-wide gene expression profiling analysis of *Leishmania major *and *Leishmania infantum *developmental stages reveals substantial differences between the two species

**DOI:** 10.1186/1471-2164-9-255

**Published:** 2008-05-29

**Authors:** Annie Rochette, Frédéric Raymond, Jean-Michel Ubeda, Martin Smith, Nadine Messier, Sébastien Boisvert, Philippe Rigault, Jacques Corbeil, Marc Ouellette, Barbara Papadopoulou

**Affiliations:** 1 Research Centre in Infectious Diseases, CHUL Research Centre and Department of Medical Biology, Faculty of Medicine, Laval University, Quebec, Canada

## Abstract

**Background:**

*Leishmania *parasites cause a diverse spectrum of diseases in humans ranging from spontaneously healing skin lesions (e.g., *L. major*) to life-threatening visceral diseases (e.g., *L. infantum*). The high conservation in gene content and genome organization between *Leishmania major *and *Leishmania infantum *contrasts their distinct pathophysiologies, suggesting that highly regulated hierarchical and temporal changes in gene expression may be involved.

**Results:**

We used a multispecies DNA oligonucleotide microarray to compare whole-genome expression patterns of promastigote (sandfly vector) and amastigote (mammalian macrophages) developmental stages between *L. major *and *L. infantum*. Seven per cent of the total *L. infantum *genome and 9.3% of the *L. major *genome were differentially expressed at the RNA level throughout development. The main variations were found in genes involved in metabolism, cellular organization and biogenesis, transport and genes encoding unknown function. Remarkably, this comparative global interspecies analysis demonstrated that only 10–12% of the differentially expressed genes were common to *L. major *and *L. infantum*. Differentially expressed genes are randomly distributed across chromosomes further supporting a posttranscriptional control, which is likely to involve a variety of 3'UTR elements.

**Conclusion:**

This study highlighted substantial differences in gene expression patterns between *L. major *and *L. infantum*. These important species-specific differences in stage-regulated gene expression may contribute to the disease tropism that distinguishes *L. major *from *L. infantum.*

## Background

*Leishmania *are protozoan parasites that cause a wide spectrum of clinical manifestations in humans, collectively referred to as leishmaniasis, ranging from self-resolving skin lesions (*L. major *and *L. mexicana*) to life-threatening visceral diseases (*L. donovani *and *L. infantum*) [1]. Leishmaniasis is endemic in 88 countries and the World Health Organization has estimated that more than 12 million people are currently infected with *Leishmania *and 350 million people are at risk of infection in tropical and subtropical regions of the world [1,2]. *Leishmania *parasites exist in two major developmental stages. In the alimentary tract of the sandfly vector, the parasites grow as extracellular flagellated promastigotes that are exposed to neutral pH and fluctuating temperatures averaging 25°C. Following the sand fly bite, the infective forms (metacyclic promastigotes) can be transferred to tissue macrophages of the mammalian host where they experience near-constant temperatures ranging from 35°C to 39°C and differentiate into aflagellated replicative amastigotes within the acidic phagolysosomal vacuoles [3]. During promastigote-to-amastigote differentiation, the parasites are subjected to drastic environmental changes, including a sharp rise in temperature, a drop in extracellular pH, an increased exposure to oxygen and nitrogen-reactive species, an intense extracellular proteolytic activity, and nutritional starvation. Several of these environmental signals trigger *Leishmania *differentiation [4-6] by activating many regulatory mechanisms affecting gene expression that result in important morphological and biochemical changes [7-10]. To date, several amastigote-specific [11-15] and promastigote-specific [16-18] genes have been identified in *Leishmania*, however, the molecular mechanisms governing developmental gene regulation in this organism warrant additional investigations.

*Leishmania *and the related *Trypanosoma *species possess unusual mechanisms of gene expression. The recent completion of the *Leishmania *spp. genomes indicates that protein-coding genes are organized as large polycistronic units [19,20]. Transcription has been postulated to initiate at strand switch regions on each chromosome [21] in the absence of defined RNA pol II promoters and typical general transcription factors. The maturation of individual mRNAs from polycistronic pre-mRNAs requires posttranscriptional control, which involves two coupled co-transcriptional RNA-processing reactions. These include *trans*-splicing where a capped RNA of ~39-nucleotides, the spliced leader RNA, is added to the 5'-terminus of all known protein-encoding RNAs, and 3'-end cleavage and polyadenylation (reviewed in [22]). Developmental gene regulation in *Leishmania *is determined posttranscriptionally mainly by sequences located in the 3'-untranslated regions (3'UTR). Several distinct 3'UTR elements have been identified among stage-specific transcripts to regulate mRNA stability/degradation and mRNA translation (reviewed in [22,23]).

The recent completion of the *L. major *and *L. infantum *genomic sequences [24] allowed studies of global gene expression throughout developmental life stages of these parasites. Global gene expression profiling using *Leishmania *microarrays with genome coverage between 22% and 97.5% highlighted that 2–9% of all genes analyzed were developmentally regulated [25-29]. The current study extends these microarray data by providing a detailed analysis of whole-genome stage- and species-specific gene expression profiles within *L. major *and *L. infantum *using a DNA oligonucleotide microarray representing the entire genomes of these two species. None of the previous DNA microarray studies has compared global gene expression profiling of promastigote (extracellular) versus amastigote (intracellular) forms of *L. infantum*, the causative agent of visceral leishmaniasis, the most severe form of the disease. Moreover, no studies to date have compared global changes in mRNA abundance during development between *Leishmania *species associated with different disease tropism (e.g. cutaneous vs. visceral leishmaniasis). The current comparative analyses revealed important differences in stage-regulated gene expression patterns between *L. major *and *L. infantum*. These species-specific differences may partly explain the distinct clinical pathologies, despite highly conserved genomes.

## Results

### Comparison of global gene expression profiles between promastigote and amastigote developmental stages of *Leishmania *spp

**Figure 1:**
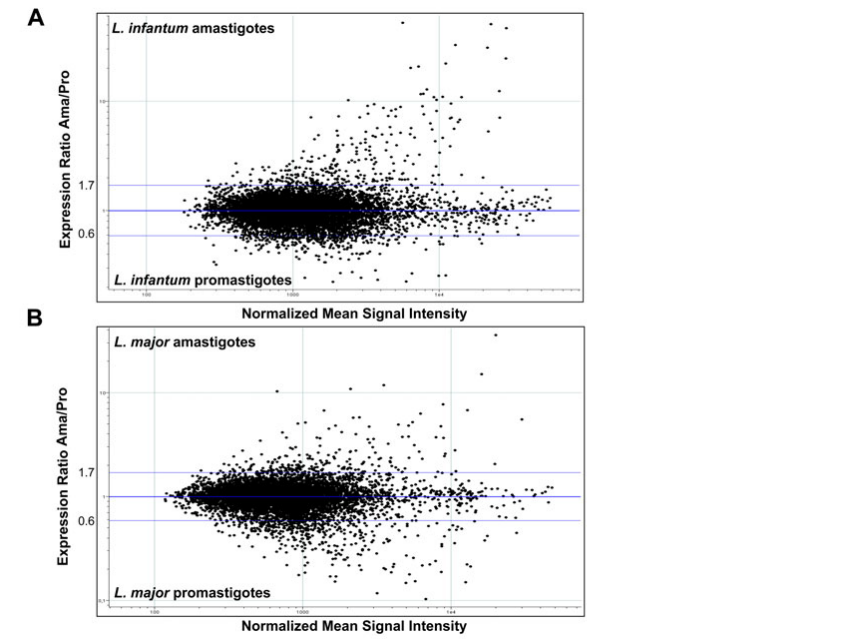
**Whole-genome expression profiling of amastigote vs. promastigote upregulated transcripts in *Leishmania *spp. **Scatter plots showing the distribution of signal intensities generated by the DNA microarray experiments employing total RNA of *L. infantum *(A) and *L. major *(B) extracted from promastigote (Pro) and amastigote (Ama) developmental life stages. The horizontal axis displays the normalized mean signal intensity for each gene (([Pro]+ [Ama]/2) and the vertical axis shows the normalized ratio of amastigote versus promastigote gene expression ([Ama]/[Pro]). External line represents a 1.7-fold change. Genes significantly upregulated in the amastigote stage are above the line at 1.7 and genes upregulated in the promastigote stage are below the line at 0.6. The data presented here are the average of six independent biological replicates for *L. infantum *and four independent biological replicates for *L. major*.

To investigate global mRNA expression profiles of *L. infantum *and *L. major *promastigote and amastigote developmental life stages, we designed a high-density multispecies 70-mer oligonucleotide genome microarray representing the entire genomes of *L. major *and *L. infantum *that share over 99% of their genes and contain species-specific genes. This allows a comparative analysis under the same conditions. Through a rigorous statistical approach, data from several independent *L. infantum *and *L. major *DNA microarray experiments were compiled and compared here. After subtracting the background, the difference of 1.7-fold in the signal intensity between the experimental conditions used (promastigote vs. amastigote RNA) for a given gene was chosen as the cut-off given that the *p *value confidence was more than 95% under those conditions. The whole-genome expression patterns of *L. infantum *(Figure 1A) and *L. major *(Fig. 1B) developmental stages are shown in the scatter plots of normalized data. The scatter plots compared each gene according to the normalized log2 ratio of the Alexa 647/Alexa 555 signal intensities (amastigotes/promastigotes) and to the signal mean intensity of each spot. More than 86% of the spots yielded hybridization intensities of two-fold over local background. Non-modulated genes, considered as constitutively expressed, had ratios between 0.6 and 1.7 (log2 = ± 0.75). Remarkably, most *Leishmania *spots yielded expression ratios close to 1 (log2 = 0), with only a few hundred genes showing modulation of greater than 1.7-fold between developmental stages in both species (Figure 1 and Table 1). The vast majority of genes (91–93%) in both *Leishmania *species were not significantly modulated (less than 1.7-fold modulation at the level of mRNA expression) throughout the two life cycle stages studied.

**Table 1:** Patterns of global differential gene expression in *Leishmania infantum *and *Leishmania major*^a^.

	*L. infantum*		*L. major*	
		
Fold increase	promastigotes	amastigotes	promastigotes	amastigotes
**1.7–3.0**	**260**	**217**	**413**	**233**
3.1–6.0	14	52	63	35
6.1–9.0	0	19	4	15
9.1–12.0	0	9	1	7
> 12.1	0	12	0	11

**Total**	**274**	**309**	**481**	**301**

**% of modulated genes**	**3.3**	**3.8**	**5.7**	**3.6**

^a ^All genes presented here (total 1365) showed a fold increase in mRNA accumulation more than 1.7 and a *p *value < 0.05.

### Comparison of global gene expression profiles of *Leishmania infantum *developmental stages

**Figure 2:**
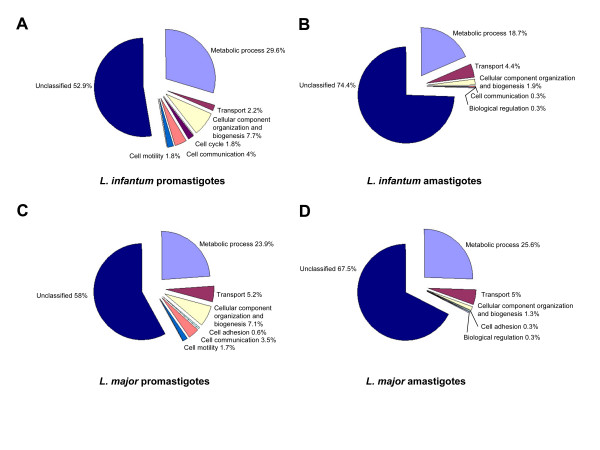
**Distribution of *Leishmania infantum *and *Leishmania major *differentially expressed genes according to Gene Ontology (GO) function categories.** GO categories for *L. infantum *promastigotes (A), *L. infantum *amastigotes (B), *L. major *promastigotes (C) and *L. major *amastigotes (D). The majority of genes encode unknown biological functions. Unclassified proteins include the hypothetical proteins (proteins with unknown function and not tested experimentally) and proteins with no GO category (unclassified) that have been experimentally characterized (e.g. amastins).

To date, no studies on the global stage-specific gene expression of a visceralizing *Leishmania *species have been reported. We therefore undertook a microarray-based approach to determine differential gene expression patterns of the two major *L. infantum *life cycle stages: (i) non-infective replicating extracellular procyclic promastigotes, and (ii) intracellular amastigotes isolated from THP-1-infected human monocytes *in vitro *following 4 days post-infection. Hybridizations were carried out using RNA from six experimental biological replicates. After normalization and data processing in order to assess differential gene expression, the data were first filtered by FDR-corrected *p*-value (*p *< 0.05) and then according to the modulation of each probe (see Methods). This analysis led to 583 genes (7.3% of the total *L. infantum *genome) showing > 1.7-fold change in mRNA abundance between the promastigote and amastigote life stages (Table 1). Analysis of the stage-modulated genes according to their gene ontology (GO) showed that they belong to several categories, including various biological processes (see Additional files 1 and 2 and Figure 2A–B).

Two hundred and seventy four genes representing 3.3% of the *L. infantum *genome were preferentially expressed in procyclic promastigotes (listed in Additional file 1). Fold-increase in mRNA abundance was modest (1.7- to 3-fold) for 95% of the overexpressed transcripts (Table 1). This group included the well-documented promastigote-regulated genes PFR1D and PFR2C [18], several dyneins and kinesins that are important for flagellar movement, cytokinesis, and motility [30,31], histones [32,33], kinetoplast DNA-associated proteins, the U-rich RNA-binding proteins UPB1 and UBP2 [34] and a member of the Pumilio family of translation repressors [35], calpain cysteine peptidases and several proteasome subunits (see Additional file 1). Half of the promastigote-upregulated genes (49%) encoded hypothetical proteins with no similarity to other known proteins in the databases.

Three hundred and nine genes, representing 3.8% of the total *L. infantum *protein-coding sequences (Table 1) were found upregulated in intracellular amastigotes compared to extracellular promastigotes (listed in Additional file 2). Transcripts modulated specifically in amastigotes showed generally a higher accumulation than promastigote-upregulated transcripts, with 31% of the transcripts demonstrating more than 3-fold increase in expression levels (average of 9.5-fold) (Table 1). Several well-documented gene families, including the amastins [15], members of the glycosylation phosphoglycan beta 1,3 galactosyltransferase gene family (SCG: Side Chain Galactose) [36] and SHERP1, a small hydrophilic protein localizing to the endoplasmic reticulum and outer mitochondrial membrane [29,37] were upregulated in *L. infantum *amastigotes. Other representative examples included the myo-inositol-1-phosphate synthase, a mRNA capping methyltransferase involved in cap4 methylation of the spliced-leader RNA [38], aminopeptidases and the hs1vu complex proteolytic subunit and several ABC, amino acid and mitochondrial transporters (see Additional file 2). Approximately 74% of the amastigote-upregulated genes encoded hypothetical or unclassified proteins for which no putative biological function has been assigned.

### Comparison of global gene expression profiles of different developmental stages within *Leishmania major*

In order to compare mRNA expression profiles between *L. infantum *and *L. major *developmental life stages, we undertook also DNA microarray experiments with *L. major*. In this study, we used the *L. major *LV39 MRHO/SU/59/P strain instead of the *L. major *Friedlin genome strain (MHOM/IL/81/Friedlin) because in our hands *L. major *LV39 was more infectious in BALB/c mice. Hybridizations were performed using RNA from four experimental biological replicates. Using the same statistical analysis applied for *L. infantum*, we identified 782 differentially expressed transcripts in *L. major *(9.3% of the total *L. major *genes) from which 481 were preferentially expressed in procyclic promastigotes (5.7%) and 301 were differentially expressed in lesion-derived amastigotes (3.6%) (Table 1). Recent microarray analyses reported that 1.5% of the *L. major *genes were differentially expressed in promastigotes and 1.4% in lesion-derived amastigotes [28]. The observed differences between the two studies may be attributed to the different *L. major *strains used or to technical issues regarding RNA preparations from mice lesions and/or to differences in the oligonucleotide genome microarray design and manipulation. The 782 identified differentially expressed genes were grouped according to GO functional categories (see Additional files 3 and 4 and Figure 2C–D). The vast majority of the transcripts enriched in *L. major *promastigotes (86%) showed a modest accumulation not exceeding 3-fold. Similarly to *L. infantum*, several *L. major *amastigote-upregulated transcripts showed in average a higher differential accumulation than the promastigote-upregulated transcripts (Table 1). Representative genes among those specifically upregulated in *L. major *promastigotes encode sugar kinases and beta-fructofuranosidases involved in carbohydrate metabolism, fatty acid elongases involved in lipid metabolism, proteins participating in nucleoside-nucleotide metabolism, amino acid, glucose and pteridine transport, electron transport, proteolysis (e.g. members of the calpain-like cysteine peptidase and peptidyl dipeptidase families), signaling (e.g. MAP and serine/threonine kinases, a PP1 Ser/Thr phosphatase, calmodulins, receptor-type adenylate cyclase a and b) and a large number of microtubule-associated proteins (see Additional file 3). This group also included several well-documented differentially expressed genes in promastigotes such as histones [26,39], the glucose transporter GT2 [14], the paraflagellar rod protein PFR1D and PFR2C [18], the leishmanolysin GP63 (also called MSP) [40,41], and the surface antigen protein 2 gene family (also known as GP46) [16,42]. More than 47% of the promastigote-upregulated transcripts encoded hypothetical proteins of unknown function (see Additional file 3). Among the *L. major *amastigote-upregulated genes are included several well-documented gene families such as amastins [15], HASPA1,2 [37], SCG5 and SCG7 which are members of the phosphoglycan beta 1,3 galactosyltransferase gene family [36], and others such as RNA-binding proteins, cathepsin L-like proteins, protein kinases, tryparedoxins, a family of novel antioxidant proteins, several amino acid and pteridine transporters and a large number (51%) of hypothetical proteins (see Additional file 4).

### Comparison of global stage-regulated gene expression between *L. infantum *and *L. major*

**Figure 3:**
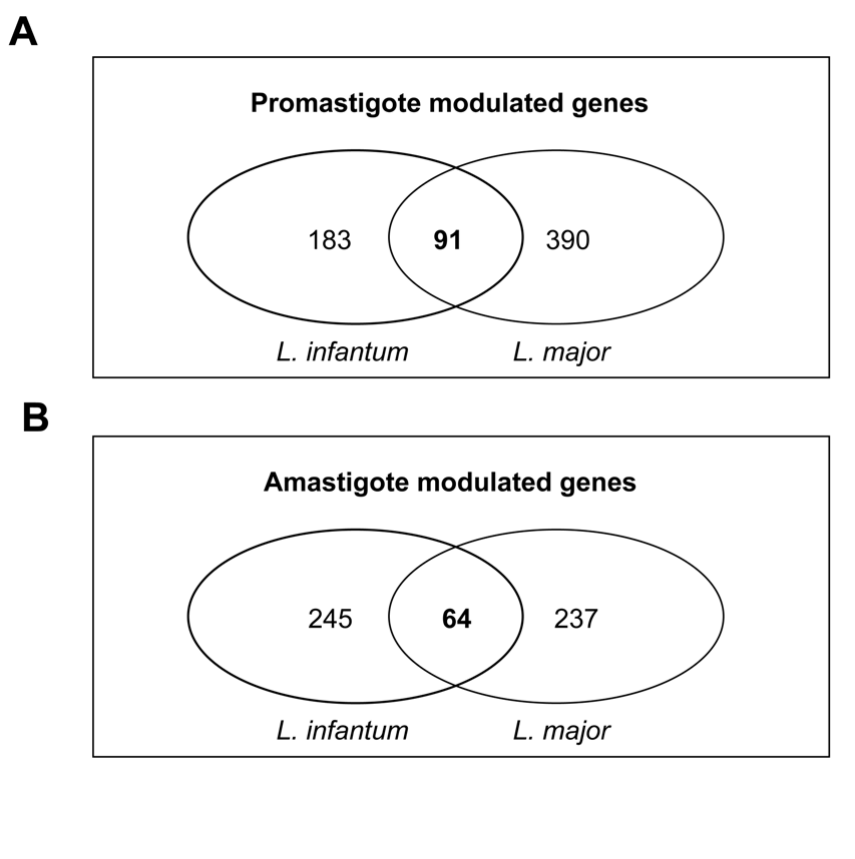
**Comparison of differential mRNA expression between *Leishmania major *and *Leishmania infantum*.** Venn diagram comparing *L. infantum *and *L. major *genes significantly upregulated (> 1.7-fold, p < 0.05) in either log-phase promastigotes or intramacrophage amastigotes. The intersection of the Venn diagrams shows the number of genes that were common to both species. These common genes are listed in Table 2. The numbers within separate circles correspond to differentially expressed genes that are unique to each species. Species comparison was performed only on probes that had less than two mismatches when hybridized to either *Leishmania *species. Thus, ~7000 probes could be directly compared between the two organisms.

One of the goals of this study was to compare global gene expression within the same life cycle stages of the *L. major *and *L. infantum *species, the causative agents of cutaneous and visceral leishmaniasis, respectively. Sequence comparison between the *L. major *and *L. infantum *genomes revealed marked conservation of synteny [20]. Therefore, much of the species-specific adaptive strategies to different target tissues and distinct disease pathogenesis should depend on the non-syntenic part of their respective genomes and/or on highly regulated hierarchical and temporal changes in gene expression. To monitor global gene expression of *L. infantum *and *L. major*, we used a multispecies high-density oligonucleotide microarray. It has recently been reported that using a multispecies microarray to study related species provided superior inter-species comparison than using several single species-specific microarrays [43]. Our comparative microarray analyses showed that out of the 755 genes differentially expressed in *L. infantum *and *L. major *promastigote forms (Figure 3) only 91 (12.05%) were in common between the two species. Similarly, only 64 (10.5%) of the 610 amastigote-upregulated genes were common to both species. These results are displayed in the form of Venn diagrams (Figure 3) and the detailed list of the commonly upregulated genes between *L. infantum *and *L. major *for a given developmental life stage is provided in Table 2. This list included, among others, the amastigote-specific amastin surface proteins, the paraflagellar rod components upregulated in promastigotes, dyneins, members of the phosphoglycan beta 1,3 galactosyltransferase, calpain-like cysteine peptidases, calmodulins and a large number of hypothetical proteins.

**Table 2:** Differentially expressed genes common to *Leishmania infantum *and *Leishmania major*.

**GO annotation (molecular function)**	**Accession number (GeneDB) *L. infantum***	**Fold increase**	**Accession number (GeneDB *L. major***	**Fold increase**
**Metabolic process**				

3-ketoacyl-coa thiolase	LinJ23_V3.0860	2,1 ^a^	LmjF23.0690	3,3 ^a^
hydrolase, alpha/beta fold family	LinJ17_V3.1110	1,7 ^a^	LmjF17.1010	1,8 ^a^
vacuolar ATP synthase subunit B	LinJ28_V3.2610	1,8 ^a^	LmjF28.2430	1,7 ^a^
succinyl-coA:3-ketoacid-coenzyme A transferase ^b^	LinJ33_V3.2470	1,8 ^c^	LmjF33.2340	2,1 ^a^
hydrolase, alpha/beta fold family	LinJ17_V3.1110	1,7 ^c^	LmjF17.1010	1,8 ^c^

**Carbohydrate metabolic process**				

acetyl-CoA synthetase	LinJ23_V3.0880	2,0 ^a^	LmjF23.0710	4,5 ^a^
aldose 1-epimerase	LinJ35_V3.1000	1,8 ^a^	LmjF35.0980	2,4 ^a^
enolase	LinJ14_V3.1240	2,3 ^a^	LmjF14.1160	2,4 ^a^
myo-inositol-1-phosphate synthetase	LinJ14_V3.1450	2,4 ^c^	LmjF14.1360	4,7 ^c^

**Lipid metabolic process**				

3-oxo-5-alpha-steroid 4-dehydrogenase	LinJ25_V3.1850	1,7 ^a^	LmjF25.1770	3,0 ^a^
fatty acid elongase	LinJ14_V3.0700	2,0 ^a^	LmjF14.0670	3,2 ^a^
lathosterol oxidase	LinJ23_V3.1560	1,9 ^a^	LmjF23.1300	3,6 ^a^
phosphoglycan beta 1,3 galactosyltransferase	LinJ02_V3.0140	1,8 ^a^	LmjF02.0160	3,8 ^a^
phosphoglycan beta 1,3 galactosyltransferase ^d^		22,0 ^c^		2,4-11,8 ^c^
hypothetical	LinJ13_V3.0200	2,7 ^c^	LmjF13.0200	3,8 ^c^

**Nucleobase, nucleoside, nucleotide and nucleic acid metabolic process**				

adenylosuccinate synthetase	LinJ13_V3.1090	1,8 ^a^	LmjF13.1190	2,0 ^a^
	LinJ25_V3.1210			
	LinJ25_V3.2580		LmjF25.1170,	
ATPase beta subunit	LinJ25_V3.2590	2,1 ^a^	LmjF25.1180	1,9 ^a^

DNA metabolic process				

3'-nucleotidase/nuclease	LinJ12_V3.0350	2,0 ^a^	LmjF12.0400	3,9 ^a^
3'-nucleotidase/nuclease precursor	LinJ31_V3.2380	1,7 ^a^	LmjF31.2310	4,6 ^a^
histone 1	LinJ33_V3.3390	2,6 a	LmjF33.3240	1,9 ^a^
3'-nucleotidase/nuclease	LinJ31_V3.2370	2,0 ^c^	LmjF31.2300	1,7 ^c^
double-strand-break repair protein rad21	LinJ05_V3.1090	3,0 ^c^	LmjF05.1090	1,9 ^c^

RNA metabolic process				

exosome complex exonuclease RRP45	LinJ22_V3.1430	3,1 ^c^	LmjF22.1580	1,8 ^c^
RNA-binding protein 5	LinJ09_V3.0080	3,4 c	LmjF09.0060	1,7 ^c^

**Amino acid and derivative metabolic process**				

glutamate dehydrogenase	LinJ15_V3.1070	1,8 ^a^	LmjF15.1010	1,8 ^a^

**Protein metabolic process**				

HSP 70	LinJ32_V3.2050	2,0 ^a^	LmjF32.1940	1,9 ^a^

Proteolysis				

	LinJ14_V3.0910,			
calpain-like cysteine peptidase	LinJ14_V3.0920	1,7 ^a^	LmjF14.0850	4,5 ^a^
calpain-like cysteine peptidase	LinJ20_V3.1320	2,0 ^a^	LmjF20.1280	2,1 ^a^
calpain-like cysteine peptidase	LinJ20_V3.1350	1,7 ^a^	LmjF20.1310	2,9 ^a^
calpain-like cysteine peptidase	LinJ27_V3.2490	1,8 ^a^	LmjF27.0510	2,8 ^a^
calpain-like cysteine peptidase	LinJ32_V3.1020	1,8 ^a^	LmjF32.0970	1,9 ^a^
			LmjF01.0830,	
			LmjF02.0740,	
peptidyl-dipeptidase	LinJ02_V3.0710	1,7 ^a^	LmjF27.2660	2,5 ^a^
puromycin-sensitive aminopeptidase-like	LinJ12_V3.0830	2,1 ^c^	LmjF12.1250	1,8 ^c^

**Protein modification process**				

protein kinase A catalytic subunit	LinJ18_V3.1090	1,9 ^a^	LmjF18.1080	3,2 ^a^
protein kinase A regulatory subunit	LinJ13_V3.0160	2,5 ^a^	LmjF13.0160	2,1 ^a^
protein kinase	LinJ30_V3.1780	2,5 ^c^	LmjF30.1780	1,7 ^c^

**Electron transport**				

trypanothione synthetase	LinJ23_V3.0500	1,7 ^c^	LmjF23.0460	1,8 ^c^

**Cellular component organization and biogenesis**				

dynein heavy chain	LinJ13_V3.1390	1,7 ^a^	LmjF13.1650	2,1 ^a^
dynein heavy chain	LinJ26_V3.1000	1,9 ^a^	LmjF26.1020	1,7 ^a^
dynein heavy chain	LinJ28_V3.0650	1,8 ^a^	LmjF28.0610	2,0 ^a^
dynein heavy chain (pseudogene)	LinJ27_V3.2460	2,1 ^a^	LmjF27.2590	2,0 ^a^
dynein light chain	LinJ24_V3.1050	2,0 ^a^	LmjF24.1030	2,0 ^a^
dynein-associated roadblock	LinJ35_V3.1740	1,8 ^a^	LmjF35.1750	2,1 ^a^
OSM3-like kinesin	LinJ17_V3.0890	1,7 ^a^	LmjF17.0800	2,0 ^a^

**Cell motility**				

			LmjF39.1750,	
	LinJ29_V3.1880,		LmjF29.1760	
PFR 1D	LinJ29_V3.1890	3,9 ^a^	LmjF29.1770	4,5 a
	LinJ16_V3.1510,		LmjF16.1425,	
PFR 2C	LinJ16_V3.1520	3,7 ^a^	LmjF16.1430	5,3 ^a^
paraflagellar rod component	LinJ09_V3.1390	1,9 ^a^	LmjF09.1320	2,8 ^a^

**Cell communication**				

			LmjF09.0910,	
	LinJ09_V3.0970,		LmjF09.0920	
calmodulin	LinJ09_V3.0980	2,0 ^a^	LmjF09.0930	2,8 ^a^
calmodulin	LinJ13_V3.1060	1,8 ^a^	LmjF13.1160	1,8 ^a^
receptor-type adenylate cyclase a	LinJ17_V3.0120	1,7 ^a^	LmjF17.0200	1,8 ^a^
			LmjF17.0230,	
	LinJ17_V3.0140,		LmjF17.0235	
receptor-type adenylate cyclase b	LinJ17_V3.0160	1,8 ^a^	LmjF17.0237	2,6 ^a^

**Transport**				

amino acid transporter aATP11 b	LinJ31_V3.0370	2,4 ^c^	LmjF31.0350	4,7 ^a^
phosphate-repressible phosphate permease	LinJ03_V3.0480	1,7 ^c^	LmjF03.0500	2,0 ^c^

**Unclassified**				

oxidoreductase	LinJ36_V3.4380	1,7 ^a^	LmjF36.4170	1,7 ^a^
leucine rich repeat protein	LinJ32_V3.3200	1,7 ^a^	LmjF32.3010	2,2 ^a^
leucine rich repeat protein	LinJ10_V3.0160	2,1 ^a^	LmjF10.0180	2,3 ^a^
protein tyrosine phosphatase	LinJ05_V3.0280	1,8 ^a^	LmjF05.0280	2,6 ^a^
hypothetical	LinJ01_V3.0640	2,4 ^a^	LmjF01.0620	2,3 ^a^
hypothetical	LinJ02_V3.0520	3,9 ^a^	LmjF02.0550	5,0 ^a^
hypothetical	LinJ07_V3.0040	2,0 ^a^	LmjF07.0030	1,8 ^a^
hypothetical	LinJ07_V3.0470	1,8 ^a^	LmjF07.0310	2,7 ^a^
hypothetical	LinJ09_V3.1620	2,1 ^a^	LmjF09.1530	1,8 ^a^
hypothetical	LinJ10_V3.1370	1,9 ^a^	LmjF10.1230	1,9 ^a^
hypothetical	LinJ11_V3.0620	2,0 ^a^	LmjF11.0610	2,1 ^a^
hypothetical	LinJ11_V3.1040	2,1 ^a^	LmjF11.1040	2,0 ^a^
hypothetical	LinJ17_V3.0970	2,0 ^a^	LmjF17.0870	3,6 ^a^
hypothetical	LinJ18_V3.1640	3,5 ^a^	LmjF18.1640	6,6 ^a^
hypothetical	LinJ19_V3.0520	4,5 ^a^	LmjF19.0520	5,9 ^a^
hypothetical	LinJ20_V3.0760	1,7 ^a^	LmjF20.0700	1,8 ^a^
hypothetical	LinJ21_V3.0440	1,8 ^a^	LmjF21.0380	2,5 ^a^
hypothetical	LinJ23_V3.1190	2,0 ^a^	LmjF23.1020	4,6 a
hypothetical	LinJ24_V3.1630	4,1 ^a^	LmjF24.1560	4,1 ^a^
hypothetical	LinJ24_V3.2200	2,4 ^a^	LmjF24.2110	1,8 ^a^
hypothetical	LinJ26_V3.2400	2,1 ^a^	Lm jF26.2380	3,0 ^a^
hypothetical	LinJ27_V3.0720	1,9 ^a^	LmjF27.0870	1,7 ^a^
hypothetical	LinJ28_V3.1150	2,1 a	LmjF28.1060	2,9 ^a^
hypothetical	LinJ29_V3.0360	1,7 ^a^	Lm jF29.0350	1,9 ^a^
hypothetical	LinJ29_V3.1090	2,4 ^a^	Lm jF29.1000	3,0 ^a^
hypothetical	LinJ29_V3.1190	2,0 ^a^	Lm jF29.1100	1,8 ^a^
hypothetical	LinJ29_V3.1260	2,5 ^a^	Lm jF29.1170	3,2 ^a^
hypothetical	LinJ29_V3.2940	2,5 ^a^	Lm jF29.2830	3,7 ^a^
hypothetical	LinJ30_V3.2870	4,4 ^a^	Lm jF30.2850	8,5 ^a^
hypothetical	LinJ31_V3.1220	2,7 ^a^	LmjF31.1200	2,0 ^a^
hypothetical	LinJ32_V3.0360	1,7 ^a^	Lm jF32.0350	2,0 ^a^
hypothetical	LinJ32_V3.0370	2,2 ^a^	Lm jF32.0360	2,2 ^a^
hypothetical	LinJ32_V3.1840	2,2 ^a^	Lm jF32.1760	3,0 ^a^
hypothetical	LinJ32_V3.2020	2,8 ^a^	Lm jF32.1910	2,4 ^a^
hypothetical	LinJ33_V3.0660	3,7 ^a^	Lm jF33.0610	5,4 a
hypothetical	LinJ34_V3.1620	2,0 ^a^	Lm jF34.1520	4,0 ^a^
hypothetical	LinJ34_V3.2590	1,7 ^a^	LmjF34.2760	1,8 ^a^
hypothetical	LinJ34_V3.4230	1,9 ^a^	Lm jF34.4600	3,0 ^a^
hypothetical	LinJ35_V3.5310	2,4 ^a^	Lm jF35.5340	4,0 ^a^
hypothetical	LinJ36_V3.0800	2,4 ^a^	LmjF36.0740	1,8 ^a^
hypothetical	LinJ36_V3.3000	1,8 ^a^	Lm jF36.2850	2,0 ^a^
hypothetical	LinJ36_V3.3780	1,8 ^a^	Lm jF36.3620	6,7 a
hypothetical	LinJ36_V3.4440	4,1 ^a^	Lm jF36.4230	5,7 ^a^
hypothetical	LinJ36_V3.5010	2,7 ^a^	Lm jF36.4780	3,2 ^a^
hypothetical	LinJ36_V3.5140	2,3 a	LmjF36.4910	1,9 ^a^
hypothetical	LinJ36_V3.5210	1,8 ^a^	LmjF36.4980	2,5 ^a^
hypothetical	LinJ35_V3.3780	1,8 ^c^	LmjF35.3730	1,7 ^a^
			LmjF 23.1050,	
	LinJ23_V3.1210,		LmjF 23.1080,	
SHERP ^b^	LinJ23_V3.1230	2,5 ^c^	LmjF23.1086	1,7 ^a^
Amastins ^e^		1.9–6.8 ^c^		1.9–25.4 ^c^
			LmjF 05.1230,	
			LmjF19.1650	
GIPL galf transferase	LinJ32_V3.4140	2,8 ^c^	LmjF32.3990	2,0 ^c^
hypothetical	LinJ01_V3.0650	1,7 ^c^	LmjF01.0630	2,5 ^c^
hypothetical	LinJ06_V3.1030	2,1 ^c^	LmjF06.0995	1,8 ^c^
hypothetical	LinJ08_V3.0650	2,0 ^c^	Lm jF08.0640	4,8 ^c^
hypothetical	LinJ10_V3.1130	2,7 ^c^	LmjF10.1050	1,8 ^c^
hypothetical	LinJ12_V3.0440	1,9 ^c^	Lm jF12.0480	5,1 ^c^
hypothetical	LinJ16_V3.0100	1,9 ^c^	Lm jF16.0090	2,5 ^c^
hypothetical	LinJ24_V3.2320	2,7 ^c^	LmjF24.2230	1,7 ^c^
hypothetical	LinJ25_V3.2870	2,3 ^c^	LmjF35.2820	1,8 ^c^
hypothetical	LinJ26_V3.1440	1,8 ^c^	Lm jF26.1460	2,0 ^c^
hypothetical	LinJ27_V3.0770	20,8 ^c^	Lm jF27.0910	1,9 ^c^
hypothetical	LinJ27_V3.2320	2,5 ^c^	LmjF27.2370	1,8 ^c^
hypothetical	LinJ30_V3.0820	2,0 ^c^	LmjF30.0770	1,8 ^c^
hypothetical	LinJ30_V3.1000	7,1 ^c^	LmjF30.0940	2,7 ^c^
hypothetical	LinJ30_V3.2340	1,7 ^c^	Lm jF30.2330	2,4 ^c^
hypothetical	LinJ31_V3.1190	2,1 ^c^	Lm jF31.1170	2,7 ^c^
hypothetical	LinJ 31_V 3.2140	1,9 ^c^	LmjF 31.2090	2,0 ^c^
hypothetical	LinJ 32_V3.3600	1,9 ^c^	LmjF 32.3400	2,3 ^c^
hypothetical	LinJ 33_V3.1720	4,2 ^c^	Lm jF 33.1620	4,5 ^c^
hypothetical	LinJ 35_V3.5130	1,7 ^c^	Lm jF 35.5160	1,8 ^c^

^a ^Genes differentially expressed in promastigotes.

^b ^Genes differentially expressed but not in the same life stage.

^c ^Genes differentially expressed in amastigotes.

^d ^The majority of the phosphoglycan 1,3 galactosyltransferase gene family members are differentially expressed in amastigotes. These genes are listed in Additional files 2 and 4.

^e ^The majority of the amastin gene family members are differentially expressed in amastigotes. These genes are listed in Additional files 2 and 4.

One hundred and nineteen genes were differentially expressed in *L. infantum *but were not modulated in *L. major *(Table 3). More than 50% of these genes encoded hypothetical proteins. For example, the LinJ29_V3.0930 gene was overexpressed 47-fold in *L. infantum *amastigotes but mRNA expression of its *L. major *orthologue remained unchanged. Other examples of genes upregulated in *L. infantum *amastigotes but not developmentally regulated in *L. major *included the aminopeptidase LinJ33_V3.2700 and metallopeptidases LinJ16_V3.0850 and LinJ34_V3.1130, the ABC transporters LinJ29_V3.0640 and LinJ11_V3.0040, the Rab GTPase activator protein LinJ29_V3.1670, the chaperone DNAJ LinJ21_V3.0550, the kinesin LinJ25_V3.2050, the mRNA capping methyltransferase LinJ36_V3.0130 and the nuclear cap binding protein LinJ30_V3.0560 (Table 3). Alternatively, 131 genes were stage-regulated in *L. major *but constitutively expressed in *L. infantum *(Table 4). Most of these *L. major *species-regulated genes were predominantly expressed in promastigotes. These included genes involved in carbohydrate metabolism like the three beta-fructofuranosidases in the *L. major *genome, 6-phospho-1-fructokinase LmjF29.2510 and hexokinase LmjF21.0250, genes participating in proteolysis such as the calpain-like cysteine peptidases LmjF20.1190 and LmjF27.0510 and carboxypeptidases LmjF13.0090 and LmjF33.2540, genes involved in electron transport such as the lactate dehydrogenase LmjF29.0280, the amino acid permease LmjF27.0680 and the transmembrane amino acid transporter LmjF07.1160 (9.7-fold of regulation), the glucose transporters lmgt2 LmjF36.6280/LmjF36.6290 and the surface antigens prostaglandin f2-alpha synthase and membrane-bound acid phosphatase 2 (Table 4). Previous studies have reported that the membrane-bound acid phosphatase 2 is a marker of virulence of *L. donovani *promastigotes [44] and that in *L. mexicana *this gene was not required for amastigote survival [45].

**Table 3:** Genes differentially expressed in *Leishmania infantum *but constitutively expressed in *Leishmania major*.

**GO annotation (molecular function)**	**Accession number (GeneDB)**	**Fold increase**	**Accession number (GeneDB)**	**Fold increase **^a^
	***L. infantum***		***L. major***	
**Metabolic process**				

glycerolphosphate mutase	LinJ33_V3.2220	2,2 ^b^	LmjF33.2100	1,4
iron superoxide dismutase	LinJ32_V3.1920	1,9 ^b^	LmjF32.1839	1,2
proteasome alpha 7 subunit	LinJ27_V3.0190	1,9 b	LmjF27.0190	1,3
pyruvate phosphate dikinase	LinJ11_V3.1000	1,8 ^b^	LmjF11.1000	1,3
nuclear receptor binding factor-like protein	LinJ05_V3.0520	2,1 ^c^	LmjF05.0520	1,3
quinone oxidoreductase	LinJ03_V3.0550	11,7 ^c^	LmjF03.0570	1,0
quinonoid dihydropteridine reductase	LinJ34_V3.4270	20,2 ^c^	LmjF34.4330	1,0

**Response to oxidative stress**				

ascorbate-dependent peroxidase	LinJ34_V3.0070	3,0 ^b^	LmjF34.0070	1,6

**Carbohydrate metabolic process**				

6-phosphogluconate dehydrogenase, decarboxylating	LinJ35_V3.3390	2,0 ^b^	LmjF35.3340	1,0
pyruvate kinase	LinJ35_V3.5450	1,9 ^b^	LmjF35.0030	1,4

**Nucleobase, nucleoside, nucleotide and nucleic acid metabolic process**				

thymidine kinase	LinJ21_V3.1450	1,7 ^b^	LmjF21.1210	1,3
DNA-directed RNA polymerase, alpha subunit	LinJ19_V3.0660	1,9 ^c^	LmjF19.0660	1,0
nuclear cap binding protein	LinJ30_V3.0560	4,1 ^c^	LmjF30.0540	1,1
vacuolar ATP synthase subunit	LinJ12_V3.0480	1,7 ^c^	LmjF12.0520	1,0

DNA metabolic process				

kinetoplast DNA-associated protein	LinJ36_V3.6180	1,8 ^b^	LmjF36.5920	1,1

RNA metabolic process				

RNA binding protein	LinJ04_V3.1190	2,2 b	LmjF04.1170	1,0
RNA binding protein UBP1	LinJ25_V3.0500	2,1 ^b^	LmjF25.0490	1,1
RNA binding protein UBP2	LinJ25_V3.0510	2,1 b	LmjF25.0500	1,1
mRNA capping methyltransferase	LinJ36_V3.0130	7,3 ^c^	LmjF36.0120	1,1
pseudouridylate synthase-like	LinJ01_V3.0280	1,9 ^c^	LmjF01.0280	1,1
RNA-binding protein	LinJ17_V3.0610	1,7 c	LmjF17.0550	1,3
RNase PH-like exosome associated protein 1	LinJ20_V3.1400	2,0 ^c^	LmjF20.1360	1,1

**Amino acid and derivative metabolic process**				

glycine dehydrogenase	LinJ26_V3.0040	8,1 ^c^	LmjF26.0030	1,4

**Protein metabolic process**				

Proteolysis				

mitochondrial processing peptidase, beta subunit	LinJ35_V3.1390	2,1 ^b^	LmjF35.1380	1,0
aminopeptidase	LinJ19_V3.0150	1,7 ^c^	LmjF19.0160	1,2
aminopeptidase	LinJ33_V3.2700	4,7 ^c^	LmjF33.2570	1,3
mitochondrial ATP-dependent zinc metallopeptidase	LinJ34_V3.1130	3,6 ^c^	LmjF34.1060	1,1

Protein folding				

chaperone protein DNAJ	LinJ18_V3.1470	1,8 ^c^	LmjF18.1490	1,1
DNAJ protein	LinJ21_V3.0550	4,5 ^c^	LmjF21.0490	1,0

**Protein modification process**				

protein kinase	LinJ27_V3.0100	4,6 ^c^	LmjF27.0100	1,0

**Electron transport**				

oxidoreductase-like protein	LinJ19_V3.1490	1,7 ^b^	LmjF19.1450	1,0
trypanothione reductase	LinJ05_V3.0350	1,7 ^b^	LmjF05.0350	1,1

**Transport**				

mitochondrial carrier protein	LinJ35_V3.3380	1,8 ^b^	LmjF35.3330	1,5
	LinJ28_V3.2050,			
zinc transporter	LinJ28_V3.2060	2,5 ^b^	LmjF28.1930	1,2
ABC transporter	LinJ29_V3.0640	4,8 ^c^	LmjF29.0620	1,6
ABC transporter	LinJ11_V3.0040	10,9 ^c^	LmjF11.0040	1,3
amino acid permease	LinJ36_V3.0450	30,9 ^c^	LmjF36.0420	1,4
pteridine transporter	LinJ06_V3.1320	2,8 ^c^	LmjF06.1260	1,6

**Cellular component organization and biogenesis**				

	LinJ16._V3.1550,			
kinesin	LinJ16_V3.1570	1,8 ^b^	LmjF16.1460	1,0
kinesin	LinJ23_V3.0720	1,9 ^b^	LmjF23.0560	1,1
kinesin	LinJ25_V3.2050	3,8 ^c^	LmjF25.1970	1,1

**Cell cycle**				

cyclin-dependent kinase regulatory subunit	LinJ32_V3.3940	1,7 ^c^	LmjF32.3790	1,0

**Cell communication**				

phosphoinositide-binding protein	LinJ35_V3.2470	1,9 ^b^	LmjF35.2420	1,2
rab11B GTPase	LinJ32_V3.1930	1,8 ^b^	LmjF32.1840	1,1

**Unclassified**				

hypothetical	LinJ02_V3.0430	4,3 b	LmjF02.0460	1,4
hypothetical	LinJ19_V3.0570	1,8 b	LmjF19.0540	1,3
hypothetical	LinJ19_V3.0610	1,8 ^b^	LmjF19.0610	1,1
hypothetical	LinJ19_V3.1150	1,9 ^b^	LmjF19.1160	1,2
hypothetical	LinJ20_V3.0030	3,6 ^b^	LmjF20.0030	1,1
hypothetical	LinJ23_V3.0010	2,1 ^b^	LmjF23.0010	1,6
hypothetical	LinJ23_V3.1170	2,2 ^b^	LmjF23.1000	1,3
hypothetical	LinJ24_V3.2420	1,8 ^b^	LmjF24.2330	1,2
hypothetical	LinJ27_V3.1110	2,5 ^b^	LmjF27.1230	1,3
hypothetical	LinJ28_V3.0100	1,8 ^b^	LmjF28.0100	1,3
hypothetical	LinJ32_V3.0530	1,7 ^b^	LmjF32.0500	1,3
hypothetical	LinJ33_V3.1200	2,6 ^b^	LmjF33.1140	1,1
hypothetical	LinJ33_V3.2570	1,8 ^b^	LmjF33.2440	1,0
hypothetical	LinJ35_V3.0180	1,8 ^b^	LmjF35.0180	1,3
hypothetical	LinJ35_V3.4360	1,7 ^b^	LmjF35.4290	1,2
hypothetical	LinJ36_V3.5520	2,9 ^b^	LmjF36.5290	1,1
A-1	LinJ29_V3.1020	2,4 ^c^	LmjF29.0935	1,6
dehydrogenase/oxidoreductase-like protein	LinJ02_V3.0700	2,0 ^c^	LmjF02.0730	1,2
EF hand-like protein	LinJ13_V3.1490	1,9 ^c^	LmjF13.1450	1,3
ferric reductase transmembrane protein	LinJ30_V3.2050	2,0 ^c^	LmjF30.2050	1,2
GTPase activator protein	LinJ29_V3.1670	7,1 ^c^	LmjF29.1560	1,1
leucine rich repeat protein	LinJ28_V3.2790	5,5 ^c^	LmjF28.2580	1,0
lipin	LinJ06_V3.0860	1,8 ^c^	LmjF06.0830	1,0
membrane-bound acid phosphatase	LinJ28_V3.2850	2,2 ^c^	LmjF28.2650	1,4
zinc-finger protein	LinJ30_V3.2350	2,4 ^c^	LmjF30.2340	1,1
metallopeptidase	LinJ16_V3.0850	8,9 ^c^	LmjF16.0850	1,3
hypothetical	LinJ01_V3.0820	2,3 ^c^	LmjF01.0800	1,0
hypothetical	LinJ03_V3.0060	3,0 ^c^	LmjF03.0070	1,3
hypothetical	LinJ04_V3.1150	7,7 c	LmjF04.1140	1,3
hypothetical	LinJ07_V3.0390	2,1 ^c^	LmjF07.0230	1,2
hypothetical	LinJ08_V3.0170	1,9 ^c^	LmjF08.0160	1,1
hypothetical	LinJ12_V3.0710	1,8 ^c^	LmjF12.1110	1,3
hypothetical	LinJ13_V3.0230	2,7 ^c^	LmjF13.0230	1,2
hypothetical	LinJ14_V3.0560	24,6 ^c^	LmjF14.0550	1,4
hypothetical	LinJ15_V3.0510	8,5 ^c^	LmjF15.0490	1,0
hypothetical	LinJ15_V3.1000	4,7 ^c^	LmjF15.0940	1,0
hypothetical	LinJ16_V3.0620	5,2 ^c^	LmjF16.0620	1,3
hypothetical	LinJ16_V3.1260	11,6 ^c^	LmjF16.1210	1,1
hypothetical	LinJ17_V3.0600	2,4 ^c^	LmjF17.0540	1,1
hypothetical	LinJ18_V3.0120	1,9 ^c^	LmjF18.0120	1,1
hypothetical	LinJ18_V3.0300	3,0 ^c^	LmjF18.0300	1,1
hypothetical	LinJ19_V3.1170	9,0 ^c^	LmjF19.1180	1,4
hypothetical	LinJ20_V3.0450	1,8 ^c^	LmjF20.0380	1,1
hypothetical	LinJ20_V3.1670	3,3 ^c^	LmjF20.1700	1,4
hypothetical	LinJ21_V3.0920	4,6 ^c^	LmjF21.0825	1,1
hypothetical	LinJ21_V3.1220	5,1 ^c^	LmjF21.0980	1,3
hypothetical	LinJ22_V3.0110	1,8 ^c^	LmjF22.0240	1,2
hypothetical	LinJ23_V3.1520	2,3 ^c^	LmjF23.1267	1,1
hypothetical	LinJ24_V3.0700	8,2 ^c^	LmjF24.0690	1,5
hypothetical	LinJ24_V3.1080	4,0 ^c^	LmjF24.1060	1,1
hypothetical	LinJ24_V3.1620	1,9 ^c^	LmjF24.1550	1,1
hypothetical	LinJ25_V3.0220	11,0 ^c^	LmjF25.0220	1,2
hypothetical	LinJ25_V3.0460	5,0 ^c^	LmjF25.0450	1,4
hypothetical	LinJ25_V3.0560	32,7 ^c^	LmjF25.0550	1,1
hypothetical	LinJ26_V3.1260	1,9 ^c^	LmjF26.1280	1,0
hypothetical	LinJ26_V3.1850	2,8 ^c^	LmjF26.1850	1,1
hypothetical	LinJ26_V3.2220	6,5 ^c^	LmjF26.2210	1,2
hypothetical	LinJ27_V3.1080	2,8 ^c^	LmjF27.1200	1,1
hypothetical	LinJ29_V3.0930	46,7 c	LmjF29.0868	1,5
hypothetical	LinJ30_V3.0160	12,4 ^c^	LmjF30.0170	1,2
hypothetical	LinJ31_V3.0490	5,4 ^c^	LmjF31.0470	1,3
hypothetical	LinJ31_V3.1630	2,2 ^c^	LmjF31.1600	1,0
hypothetical	LinJ32_V3.1410	2,2 ^c^	LmjF32.1350	1,1
hypothetical	LinJ32_V3.3760	2,9 ^c^	LmjF32.3600	1,2
hypothetical	LinJ33_V3.0590	1,8 ^c^	LmjF33.0565	1,3
hypothetical	LinJ33_V3.0810	2,1 ^c^	LmjF33.0760	1,1
hypothetical	LinJ33_V3.2850	1,9 ^c^	LmjF33.2710	1,4
hypothetical	LinJ34_V3.2250	2,0 ^c^	LmjF34.2473	1,2
hypothetical	LinJ34_V3.3520	4,1 ^c^	LmjF34.3730	1,1
hypothetical	LinJ35_V3.4090	10,3 ^c^	LmjF35.4040	1,2
hypothetical	LinJ35_V3.1640	9,4 ^c^	LmjF35.1640	1,2
hypothetical	LinJ35_V3.5350	2,1 ^c^	LmjF35.5030	1,0
hypothetical	LinJ36_V3.0470	6,3 ^c^	LmjF36.0440	1,1
hypothetical	LinJ36_V3.4410	3,5 ^c^	LmjF36.4200	1,6
hypothetical	LinJ36_V3.4940	1,8 ^c^	LmjF36.4710	1,1

^a ^Only non-modulated genes are listed in this column.

^b ^Genes differentially expressed in promastigotes.

^c ^Genes differentially expressed in amastigotes.

**Table 4:** Genes differentially expressed in *Leishmania major *but constitutively expressed in *Leishmania infantum*.

**GO annotation (molecular function)**	**Accession number (GeneDB)**	**Fold increase **^a^	**Accession number (GeneDB)**	**Fold increase**
	***L. infantum***		***L. major***	
**Metabolic process**				

2-aminoethylphosphonate: pyruvateaminotransferase	LinJ03_V3.0400	1,0	LmjF03.0040	2,0 ^b^
folylpolyglutamate synthetase	LinJ36_V3.2740	1,1	LmjF36.2610	2,1 ^b^
phosphatidylethanolaminen-methyltransferase	LinJ31_V3.3250	1,3	LmjF31.3120	2,4 ^b^
aldehyde dehydrogenase	LinJ25_V3.1160	1,1	LmjF25.1120	3,7 c
arginase	LinJ35_V3.1490	1,3	LmjF35.1480	2,8 ^c^

**Carbohydrate metabolic process**				

6-phospho-1-fructokinase	LinJ29_V3.2620	1,4	LmjF29.2510	2,6 ^b^
aldose 1-epimerase	LinJ35_V3.0990	1,1	LmjF35.0970	3,2 ^b^
beta-fructofuranosidase	LinJ04_V3.0300	1,3	LmjF04.0310	5,4 ^b^
beta-fructofuranosidase	LinJ04_V3.0310	1,2	LmjF04.0320	3,6 ^b^
	LinJ35_V3.0640,			
beta-fructofuranosidase	LinJ35_V3.0650	1,6	LmjF35.0640	5,9 ^b^
	LinJ21_V3.0300,		LmjF21.0240,	
hexokinase	LinJ21_V3.0310	1,2	LmjF21.0250	2,2 ^b^
glyceraldehyde 3-phosphate dehydrogenase	LinJ36_V3.2480	1,4	LmjF36.2350	3,6 ^c^

**Lipid metabolic process**				

farnesyl pyrophosphate synthase	LinJ22_V3.1210	1,2	LmjF22.1360	1,9 b
fatty acid elongase	LinJ14_V3.0670	1,6	LmjF14.0650	2,7 ^b^
mevalonate kinase	LinJ31_V3.0580	1,1	LmjF31.0560	2,1 ^b^
phospholipase c-like	LinJ30_V3.2970	1,3	LmjF30.2950	2,1 ^c^
polyprenyl synthase	LinJ19_V3.0210	1,0	LmjF19.0220	2,4 c

**Nucleobase, nucleoside, nucleotide and nucleic acid metabolic process**				

adenosine deaminase	LinJ35_V3.2200	1,1	LmjF35.2160	4,6 ^b^

DNA metabolic process				

j-binding protein	LinJ14_V3.0040	1,4	LmjF14.0040	2,3 b

RNA metabolic process				

RNA-binding protein	LinJ23_V3.0900	1,3	LmjF23.0730	3,6 ^c^

**Amino acid and derivative metabolic process**				

tyrosine aminotransferase	LinJ36_V3.2490	1,1	LmjF36.2360	2,7 b

**Protein metabolic process**				

lipophosphoglycan biosynthetic protein	LinJ29_V3.0790	1,3	LmjF29.0760	2,3 ^b^

Proteolysis				

calpain-like cysteine peptidase	LinJ20_V3.1230	1,1	LmjF20.1190	2,6 ^b^
calpain-like cysteine peptidase	LinJ27_V3.0510	1,3	LmjF27.0510	2,8 ^b^
carboxypeptidase	LinJ33_V3.2670	1,6	LmjF33.2540	3,0 ^b^
carboxypeptidase	LinJ13_V3.0090	1,1	LmjF13.0090	2,1 ^b^
glutamamyl carboxypeptidase	LinJ29_V3.1680	1,1	LmjF29.1570	1,7 ^b^
oligopeptidase b	LinJ09_V3.0820	1,1	LmjF09.0770	2,2 ^b^
pyroglutamyl-peptidase (PGP)	LinJ34_V3.1750	1,4	LmjF34.2000	2,0 ^b^
cysteine protease	LinJ19_V3.1460	1,3	LmjF19.1420	3,1 ^c^

Protein folding				

chaperone protein DNAJ	LinJ32_V3.3220	1,1	LmjF32.3030	2,2 ^b^

**Protein modification process**				

protein kinase	LinJ26_V3.2600	1,2	LmjF26.2570	4,3 ^b^
protein kinase	LinJ21_V3.0190	1,1	LmjF21.0130	2,2 ^b^
mitogen-activated protein kinase 3	LinJ10_V3.0540	1,0	LmjF10.0490	2,0 ^b^
serine/threonine-protein kinase	LinJ31_V3.3070	1,1	LmjF31.2960	2,8 ^b^
serine/threonine-protein phosphatase PP1	LinJ34_V3.0850	1,1	LmjF34.0810	1,8 ^b^
protein kinase	LinJ36_V3.4460	1,6	LmjF36.4250	1,7 ^c^

**Electron transport**				

D-lactate dehydrogenase	LinJ29_V3.0290	1,1	LmjF29.0280	3,0 ^b^
pyrroline-5-carboxylate reductase	LinJ13_V3.1420	1,6	LmjF13.1680	2,1 ^b^
UDP-galactopyranose	LinJ18_V3.0200	1,1	LmjF18.0200	1,7 ^b^

**Transport**				

amino acid permease	LinJ27_V3.0530	1,6	LmjF27.0680	3,0 b
biopterin transporter	LinJ35_V3.5120	1,1	LmjF35.5150	2,4 ^b^
			LmjF36.6280,	
glucose transporter, lmgt2	LinJ36_V3.6550	1,0	LmjF36.6290	5,6 ^b^
pteridine transporter	LinJ06_V3.0310	1,1	LmjF06.0310	3,2 ^b^
sugar transporter	LinJ24_V3.0690	1,0	LmjF24.0680	3,9 ^b^
transmembrane amino acid transporter	LinJ07_V3.1340	1,1	LmjF07.1160	9,7 ^b^
	LinJ15_V3.1230,			
	LinJ15_V3.1240,			
	LinJ15_V3.1250,		LmjF15.1230,	
nucleoside transporter 1	LinJ15_V3.1260	1,2	LmjF15.1240	4,5 ^b^
mitochondrial ornithine transporter 1-like	LinJ16_V3.0220	1,0	LmjF16.0210	2,2 ^b^

**Cell component organization and biogenesis**				

dynein heavy chain	LinJ36_V3.1010	1,6	LmjF36.0950	2,6 ^b^
kinesin	LinJ14_V3.0870	1,1	LmjF14.0810	1,8 ^b^
kinesin	LinJ21_V3.1280	1,4	LmjF21.1040	1,9 ^b^
			LmjF09.0150,	
			LmjF09.0154,	
			LmjF09.0158,	
			LmjF09.0162,	
			LmjF09.0166,	
			LmjF09.0170,	
microtubule associated protein	LinJ09_V3.0180	1,3	LmjF09.0174	2,5 ^b^
	LinJ19_V3.0820,			
microtubule associated protein	LinJ19_V3.0850	1,1	LmjF19.0860	2,5 ^b^
PFR	LinJ05_V3.0920	1,3	LmjF05.0920	2,3 ^b^
PFR par4	LinJ05_V3.0040	1,3	LmjF05.0040	2,8 ^b^

**Cell communication**				

cAMP specific phosphodiesterase	LinJ15_V3.1550	1,5	LmjF15.1480	1,8 ^b^
phosphodiesterase	LinJ18_V3.1100	1,4	LmjF18.1090	2,3 ^b^

**Cell motility**				

myosin heavy chain	LinJ32_V3.4020	1,6	LmjF32.3870	3,5 ^b^

**Unclassified**				

acid phophatase	LinJ36_V3.2600	1,0	LmjF36.2470	1,9 b
long chain fatty Acyl CoA synthetase	LinJ03_V3.0220	1,1	LmjF03.0230	1,9 b
	LinJ19_V3.1350,		LmjF19.1340,	
glycerol uptake protein	LinJ19_V3.1360	1,3	LmjF19.1345	1,8 ^b^
membrane-bound acid phosphatase 2	LinJ36_V3.2720	1,1	LmjF36.2590	4,6 ^b^
nons pecific nucleoside hydrolas e	LinJ18_V3.1570	1,1	LmjF18.1580	2,1 ^b^
prostaglandin f2-alpha synthase	LinJ31_V3.2210	1,5	LmjF31.2150	5,1 ^b^
	LinJ31_V3.0950,		Lm jF 31.0920,	
s odium s tibogluconate res is tance protein	LinJ31_V3.3400	1,4	LmjF31.0950	2,1 b
s urfac e antigen-lik e	LinJ04_V3.0170	1,0	Lm jF04.0180	2,0 ^b^
s urfac e antigen-lik e	LinJ04_V3.0180	1,0	Lm jF04.0190	2,7 ^b^
			Lm jF 12.0850,	
			Lm jF 12.0860,	
			Lm jF 12.0870,	
surface antigen 2	LinJ12_V3.0020	1,2	LmjF12.0890	2,1 ^b^
ubiquitin-conjugating enzy me-like	LinJ21_V3.0500	1,6	Lm jF21.0440	2,2 ^b^
hypothetical	LinJ03_V3.0340	1,4	Lm jF03.0360	1,9 ^b^
			Lm jF 04.0130,	
			Lm jF 04.0140,	
			Lm jF 04.0150,	
			Lm jF 04.0160,	
hypothetical	LinJ04_V3.0160	1,1	LmjF04.0170	2,9 ^b^
hypothetical	LinJ05_V3.1070	1,3	Lm jF05.1070	3,0 ^b^
hypothetical	LinJ09_V3.1360	1,0	Lm jF09.1300	2,0 ^b^
hypothetical	LinJ09_V3.1600	1,4	Lm jF09.1510	2,9 ^b^
hypothetical	LinJ09_V3.1610	1,1	Lm jF09.1520	3,2 ^b^
			Lm jF 11.0670,	
	LinJ11_V3.0680,		Lm jF 11.0673,	
hypothetical	LinJ11_V3.0690	1,0	LmjF11.0675	3,0 b
hypothetical	LinJ04_V3.1220	1,3	Lm jF04.1200	2,0 ^b^
	LinJ14_V3.0490,		Lm jF 14.0480,	
hypothetical	LinJ14_V3.0500	1,3	LmjF14.0490	2,6 ^b^
hypothetical	LinJ15_V3.0560	1,0	Lm jF15.0540	1,9 ^b^
hypothetical	LinJ17_V3.0690	1,4	Lm jF17.0630	2,5 ^b^
hypothetical	LinJ17_V3.0990	1,1	Lm jF17.0890	6,5 ^b^
hypothetical	LinJ18_V3.1300	1,1	Lm jF18.1320	1,9 ^b^
hypothetical	LinJ19_V3.0070	1,4	Lm jF19.0080	3,5 ^b^
hypothetical	LinJ23_V3.0890	1,3	Lm jF23.0720	2,4 ^b^
hypothetical	LinJ23_V3.1020	1,1	Lm jF23.0840	2,6 ^b^
hypothetical	LinJ23_V3.1730	1,4	Lm jF23.1690	1,8 ^b^
hypothetical	LinJ24_V3.1110	1,1	Lm jF24.1090	4,6 ^b^
hypothetical	LinJ25_V3.2090	1,1	Lm jF25.2010	2,7 ^b^
hypothetical	LinJ26_V3.1980	1,5	Lm jF26.1980	1,9 ^b^
hypothetical	LinJ28_V3.0220	1,1	LmjF28.0220	1,7 ^b^
hypothetical	LinJ28_V3.0420	1,2	LmjF28.0280	1,8 ^b^
hypothetical	LinJ29_V3.1820	1,0	Lm jF29.1690	2,8 ^b^
hypothetical	LinJ29_V3.2550	1,6	Lm jF29.2440	3,3 ^b^
hypothetical	LinJ30_V3.1230	1,1	Lm jF30.1170	2,5 ^b^
hypothetical	LinJ30_V3.2700	1,3	Lm jF30.2700	3,3 b
hypothetical	LinJ32_V3.0480	1,3	LmjF32.0470	2,0 ^b^
hypothetical	LinJ32_V3.1760	1,4	Lm jF32.1680	3,3 ^b^
hypothetical	LinJ32_V3.3350	1,3	Lm jF32.3150	2,3 ^b^
hypothetical	LinJ32_V3.3650	1,3	Lm jF32.3450	1,9 ^b^
hypothetical	LinJ33_V3.0220	1,6	Lm jF33.0210	4,0 ^b^
hypothetical	LinJ33_V3.1040	1,0	Lm jF33.0990	3,4 ^b^
hypothetical	LinJ33_V3.1130	1,4	LmjF33.1070	2,7 ^b^
hypothetical	LinJ33_V3.2940	1,4	LmjF33.2800	1,7 ^b^
hypothetical	LinJ34_V3.0210	1,1	LmjF34.0190	2,1 ^b^
hypothetical	LinJ34_V3.0740	1,2	LmjF34.0705	1,8 ^b^
hypothetical	LinJ34_V3.4110	1,0	LmjF34.4280	2,4 ^b^
hypothetical	LinJ35_V3.1470	1,0	LmjF35.1460	1,7 ^b^
hypothetical	LinJ35_V3.5400	1,2	LmjF35.5080	1,7 ^b^
hypothetical	LinJ36_V3.0730	1,0	LmjF36.0670	2,5 ^b^
hypothetical	LinJ36_V3.1190	1,0	LmjF36.1130	1,7 ^b^
hypothetical	LinJ36_V3.1230	1,3	LmjF36.1170	2,1 ^b^
hypothetical	LinJ36_V3.1520	1,1	LmjF36.1460	1,7 ^b^
hypothetical	LinJ36_V3.4470	1,1	LmjF36.4260	2,0 ^b^
			Lm jF30.1410,	
			Lm jF30.1420,	
ama 1	LinJ30_V3.1490	1,1	LmjF30.1430	2,1 ^c^
			Lm jF31.1450,	
s urfac e membrane protein gp46-like	LinJ31_V3.1490	1,1	LmjF31.1460	2,1 ^c^
hypothetical	LinJ06_V3.0030	1,1	LmjF06.0030	2,0 ^c^
hypothetical	LinJ17_V3.0390	1,3	LmjF17.0340	4,0 ^c^
hypothetical	LinJ18_V3.1110	1,1	LmjF18.1100	1,7 c
hypothetical	LinJ19_V3.1540	1,1	LmjF19.1490	1,8 ^c^
hypothetical	LinJ20_V3.1630	1,1	LmjF20.1660	2,0 ^c^
hypothetical	LinJ24_V3.0590	1,2	LmjF24.0580	4,8 ^c^
hypothetical	LinJ25_V3.0230	1,3	LmjF25.0230	2,0 ^c^
hypothetical	LinJ26_V3.1500	1,3	LmjF26.1520	3,5 ^c^
hypothetical	LinJ28_V3.1070	1,4	LmjF28.0980	2,6 ^c^
hypothetical	LinJ28_V3.2860	1,2	LmjF28.2660	2,0 ^c^
hypothetical	LinJ31_V3.2910	1,4	LmjF31.2810	2,1 ^c^
hypothetical	LinJ32_V3.2620	1,3	LmjF32.2480	1,8 ^c^
hypothetical	LinJ35_V3.4870	1,3	LmjF35.4810	1,8 ^c^
hypothetical	LinJ36_V3.2960	1,1	LmjF36.2820	1,9 ^c^
hypothetical	LinJ36_V3.3480	1,3	LmjF36.3320	1,7 ^c^
hypothetical	LinJ36_V3.4870	1,0	LmjF36.4640	2,0 ^c^

^a ^Only non-modulated genes are listed in this column.

^b ^Genes differentially expressed in promastigotes.

^c ^Genes differentially expressed in amastigotes.

### Confirmation of gene expression patterns by quantitative real-time PCR

Quantitative real-time PCR (qRT-PCR) was used to validate the microarray results. Changes in expression levels of 80 selected *L. infantum *and/or *L. major *mRNAs in either promastigotes or intracellular amastigotes as determined by the microarray experiments were confirmed by qRT-PCR. Genes for qRT-PCR were randomly selected based on different criteria, which included high vs. lower levels of expression, constitutive vs. stage-regulated gene expression, different GO categories or genes encoding unclassified proteins, single copy genes and genes belonging to larger gene families. mRNA expression levels determined by qRT-PCR were normalized to three *L. infantum *and *L. major *constitutively expressed mRNAs (see Methods). Estimated expression patterns by qRT-PCR were compared to those by DNA microarrays. Results obtained by qRT-PCR were consistent with the microarray data in more than 97% of the cases (Table 5). In few cases (~10%), the difference in expression levels between the two methods (e.g. DNA microarray vs. qRT-PCR) was two-fold or higher, however, both methods agreed on the differential gene expression in the same life cycle stage (Table 5).

**Table 5:** Comparison of microarray mRNA expression levels to relative expression levels determined by quantitative real time PCR (qRT-PCR).

Accession number (GeneDB)	Ratio Ama/Pro	
	
	DNA Microarrays ^a^	qRT-PCR ^b^
***L. infantum***		

LinJ30_V 3.2870	0,2	0,5 ± 0,0
LinJ34_V 3.0070	0,3	0,5 ± 0,0
LinJ36_V 3.5620	0,4	0,3 ± 0,0
LinJ21_V 3.1490	0,4	0,5 ± 0,1
LinJ14_V 3.1240	0,4	0,5 ± 0,1
LinJ23_V 3.0860	0,5	0,3 ± 0,0
LinJ25_V 3.0500	0,5	0,2 ± 0,2
LinJ16_V 3.0950	0,5	0,5 ± 0,0
LinJ18_V 3.1090	0,5	0,6 ± 0,0
LinJ24_V 3.0870	0,6	0,5 ± 0,0
LinJ30_V 3.1520	1,7	2,2 ± 0,3
LinJ33_V 3.2470	1,8	1,3 ± 0,1
LinJ34_V 3.1730	2,0	2,3 ± 0,3
LinJ30_V 3.2050	2,0	1,8 ± 0,1
LinJ34_V 3.1160	2,3	1,9 ± 0,6
LinJ31_V 3.0370	2,4	2,2 ± 0,3
LinJ14_V 3.1450	2,4	3,7 ± 0,3
LinJ34_V 3.1020 ^c^	2,8	10,1 ± 0,5
LinJ30_V 3.0560	4,1	3,3 ± 0,3
LinJ27_V 3.0100	4,6	3,7 ± 0,4
LinJ19_V 3.0420	5,0	3,0 ± 0,3
LinJ36_V 3.6530	5,0	1,9 ± 0,2
LinJ23_V 3.1790	7,0	2,4 ± 0,5
LinJ36_V 3.0130	7,3	4,2 ± 0,3
LinJ31_V 3.2790	7,6	2,0 ± 0,1
LinJ26_V 3.0040	8,1	2,4 ± 0,2
LinJ36_V 3.4180	8,7	6,1 ± 0,6
LinJ34_V 3.4270	20,2	2,4 ± 0,2
LinJ14_V 3.1500	22,0	1,2 ± 0,1
LinJ14_V 3.0560	24,6	2,0 ± 0,0
LinJ36_V 3.2480	0,7	0,6 ± 0,3
LinJ14_V 3.0760	0,9	1,5 ± 0,1
LinJ25_V 3.1160	0,9	1,0 ± 0,1
LinJ07_V 3.0550	1,0	1,6 ± 0,2
LinJ28_V 3.0470	1,0	1,2 ± 0,1
LinJ34_V 3.1910	1,0	1,0 ± 0,5
LinJ19_V 3.0010	1,2	1,6 ± 0,1
LinJ34_V 3.2420	1,2	1,3 ± 0,1
LinJ30_V 3.2200	1,3	1,3 ± 0,1
LinJ36_V 3.4000	1,4	1,8 ± 0,3
LinJ17_V 3.0440	1,5	3,0 ± 0,2
LinJ08_V 3.1220	1,6	1,7 ± 0,1

***L. major***		

Lm jF33.2340	0,5	0,3 ± 0,0
Lm jF36.1360	0,5	0,8 ± 0,0
Lm jF29.2510	0,4	0,02 ± 0,00
Lm jF36.2360	0,4	0,4 ± 0,0
Lm jF06.0310	0,3	0,4 ± 0,0
Lm jF35.0970	0,3	0,6 ± 0,0
Lm jF23.0690	0,3	0,2 ± 0,0
Lm jF23.1300	0,3	0,1 ± 0,0
Lm jF02.0160	0,3	0,1 ± 0,0
Lm jF23.0710	0,2	0,1 ± 0,0
Lm jF35.2160	0,2	0,1 ± 0,0
Lm jF36.2590	0,2	0,1 ± 0,0
Lm jF21.0240	0,2	0,2 ± 0,0
Lm jF31.0350	0,2	0,1 ± 0,0
Lm jF04.0310	0,2	0,3 ± 0,0
Lm jF07.1160	0,1	0,1 ± 0,0
Lm jF31.2460	2,6	2,3 ± 0,2
Lm jF28.2910	2,8	1,9 ± 0,2
Lm jF36.5960	3,1	2,0 ± 2,1
Lm jF23.0730	3,6	1,1 ± 0,1
Lm jF36.2350	3,6	4,5 ± 0,4
Lm jF25.1120	3,7	2,3 ± 0,3
Lm jF08.0820	4,3	6,6 ± 0,9
Lm jF14.1360	4,7	4,4 ± 0,4
Lm jF30.2190	5,8	3,0 ± 0,1
Lm jF11.1220	6,7	5,2 ± 0,3
Lm jF34.1840	7,3	3,1 ± 0,2
Lm jF35.4230	10,3	1,9 ± 0,1
Lm jF31.3190	10,9	3,7 ± 0,4
Lm jF36.3810	1,0	1,1 ± 0,1
Lm jF28.0330	1,1	1,3 ± 0,2
Lm jF30.2050	1,2	1,5 ± 0,2
Lm jF03.0570	1,0	1,2 ± 0,2
Lm jF34.4510	1,0	2,0 ± 0,2
Lm jF34.0070	1,6	1,0 ± 0,1

^a ^The microarray results have all a *p*-value lower than 0.05.

^b ^The same RNA preparations were used for the microarray and qRT-PCR experiments. Levels of mRNA determined by qRT-PCR were normalized as described in Methods. The values reported here are the average of two biological replicates and three technical replicates. The set of primers for qRT-PCR amplification are presented in Additional file 6.

^c ^LinJ34_V3.1020 encodes an amastin homologue that shares homology to other members of the amastin gene family. For the microarray experiment, the 70-mer probe used recognizes more than one amastin members whereas the set of primers used for qRT-PCR was specific to the LinJ34_V3.1020 gene only.

^d ^LinJ14_V3.1500 is a member of the glycosylation phosphoglycan beta 1,3 galactosyltransferase gene family. For the microarray experiment the 70-mer probe used recognizes more than one gene family members whereas the set of primers used for qRT-PCR was specific to the LinJ14_V3.1500 gene only, whose expression was not modulated.

In this study, *L. infantum *amastigotes were isolated from THP1-infected macrophages and *L. major *amastigotes from mouse lesions. Our attempts to isolate sufficient amastigote RNA material from the spleen/liver of infected hamsters were unsuccessful due to low infection rates obtained with the *L. infantum *strain we used in this study. To investigate whether gene expression levels could be different in lesion- vs. THP1-derived amastigotes, we first evaluated by qRT-PCR analysis the expression patterns of a selected number of transcripts between *L. major *amastigotes isolated from mouse lesions or THP1 infected cells in vitro. The majority of the genes tested (73%) were modulated in a similar fashion in THP1-derived or lesion-derived *L. major *amastigotes (see Additional file 5). Even when *L. major *or *L. infantum *amastigotes were grown within THP1-infected cells, different expression patterns were obtained for the majority (67%) of the transcripts analyzed by qRT-PCR (see Additional file 5). These results further support our microarray data demonstrating substantial differences in amastigote-regulated gene expression between *L. major *and *L. infantum *(Figure 3).

### Stage-regulated transcripts are distributed throughout the *Leishmania *spp. chromosomes

**Figure 4:**
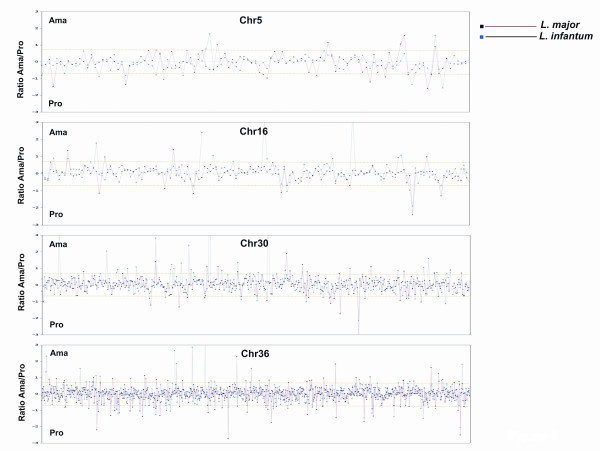
**Distribution of differentially expressed transcripts in chromosomes 5, 16, 30 and 36 of *Leishmania major *and *Leishmania infantum*.** On the horizontal axis are all genes in the order found on the selected chromosomes according to GeneDB [24]. On the vertical axis is the amastigote (Ama) to promastigote (Pro) expression ratio. Genes upregulated in the amastigote stage are above the line at 0.75 and genes upregulated in promastigotes are below the line -0.75. Blue dots represent stage-regulated expression of genes on the respective *L. infantum *chromosomes and black dots represent differential expression of genes on the same chromosomes of *L. major*.

As mentioned in the Introduction, up to several hundred *Leishmania *genes are co-transcribed into polycistronic RNAs, and individual mature mRNAs are resolved by *trans*-splicing and polyadenylation [22,23]. To determine genome distribution and chromosomal organization of the differentially expressed genes in both *Leishmania *species, global RNA expression profiles for both developmental stages of *L. major *and *L. infantum *were generated for each of the 36 chromosomes (GeneDB, [19,20]). As representative examples, only chromosomes 5, 16, 30 and 36 are shown here (Figure 4). This global genome analysis revealed that differentially expressed genes in either life stage are randomly distributed throughout *Leishmania *chromosomes and that there is apparently no clustering of these genes within specific genomic loci (Figure 4 and data not shown). These observations are consistent with published work to date and further support the concept that stage-regulated gene expression in *Leishmania *involves mostly post-transcriptional mechanisms.

### SIDER retroposons and their distribution in the 3'UTR of *Leishmania *differentially expressed transcripts

We recently identified two large classes of distinct short interspersed degenerate retroposons, named SIDER1 (~785 copies) and SIDER2 (~1073 copies) that are mainly located in the 3'UTR of *Leishmania *mRNAs [46]. Members of the SIDER1 subfamily correspond to the previously identified ~450 nt 3'UTR element conserved in several developmentally regulated mRNAs, including the amastin mRNAs [15,47]. SIDER1 was shown to regulate amastin mRNA translation in a stage-specific manner [47,48] whereas SIDER2 plays a rather global role in mRNA degradation [46]. Since SIDERs and especially SIDER2 are involved in the regulation of mRNA stability, we investigated whether these widespread retroposons were associated more frequently with differentially expressed mRNAs. Therefore, we screened all differentially expressed transcripts as determined by microarray experiments in both *Leishmania *species for the presence of SIDER1 or SIDER2 homologous sequences in their potential 3'UTR (putative 3'UTRs were mapped using bioinformatics tools as described [46,49]). On average, 21% of the promastigote-upregulated transcripts and 26–42% of the amastigote-upregulated transcripts in either species contained either SIDER1 or SIDER2 in their 3'UTR (Table 6). The higher percentage of SIDER1 in amastigotes can be partly explained by the presence of this subclass of retroposons in amastin mRNAs that are part of a large gene family ([15], unpublished data)). Interestingly, *L. major *amastigote-upregulated transcripts contained generally a higher percentage of SIDER1/2 retroposons and especially SIDER1 compared to *L. infantum *(42% vs. 26%). Considering that ~25% of the *L. major *or *L. infantum *transcripts bear either SIDER1 or SIDER2 in their 3'UTR (Table 6) ([46] (Smith, M. *et al*., unpublished)), our data indicate that SIDERs, at least in *L. infantum*, are not preferentially associated with differentially upregulated transcripts, which suggests that additional regulatory elements may also contribute to the stage-specific accumulation of *Leishmania *mRNAs.

**Table 6:** Distribution of SIDER retroposons among differentially expressed *Leishmania *spp. transcripts.

	**Differentially Expressed Transcripts**^a^			**Genomic Distribution**^b^	
		
**Species**	**Life Stage**	**SIDER1%**	**SIDER2%**	**SIDER1%**	**SIDER2%**
*L. major*	amastigote	23.9	18.3	11.6	15.0
	promastigote	10.6	11.6		
*L. infantum*	amastigote	14.1	11.9	9.4	15.8
	promastigote	8.4	12.4		

^a ^As determined by the DNA microarray experiments (this study).

^b ^Refined species-specific hidden Markov model profiles were used to determine the genomic distribution of two SIDER families in *L. infantum *and *L. major *([46]; Smith *et al*. unpublished)). Values represent the proportion of annotated coding sequences that potentially harbor at least one SIDER element in their 3'UTR. The percentage of SIDERs in differentially expressed transcripts is compared with the genomic distribution of SIDERs.

## Discussion

This study provides an extensive analysis of genome-wide expression profiling of the main developmental life stages (e.g. procyclic promastigotes and intracellular amastigotes) of *L. infantum *along with a comparative analysis of gene expression profiles between *L. infantum *and *L. major, *two species causing distinct pathologies in humans, using a multispecies *Leishmania *DNA microarray. The comparative analysis between *L. major *and *L. infantum *transcriptomes is the first to date and may lead to a better understanding of how differential gene expression among species with high similarity in genome sequences may be involved in the development of different disease phenotypes.

### Expression patterns, stage-regulated genes and pathways identified

Genes whose expression was upregulated in either promastigote or amastigote life stages of *L. major *and *L. infantum, *as determined by DNA microarrays, belong to various biological processes. Approximately 25% of the differentially expressed genes between both life stages in both species are involved in metabolism (Figure 2). Promastigotes and amastigotes reside in different environments and it is therefore expected that their metabolic needs will differ. Promastigotes in the sand fly use glycolysis and mitochondrial metabolism as their main energy sources (reviewed in [50]). Amastigotes contain glycosomes, although considerably fewer than promastigotes [50]. *L. mexicana *amastigotes isolated from mouse lesions have a reduced need for proline and glucose consumption and increased beta-oxidation of fatty acids compared to promastigotes, which could be used as energy source [51,52]. Our study revealed indeed that genes involved in carbohydrate metabolism as well as several glucose transporters were overexpressed in promastigotes compared to amastigotes (Table 2, see Additional files 1 and 1). Interestingly, recent proteomic studies using axenic *L. donovani *promastigote and amastigote cultures indicated that the differentiating parasite shifts from glucose to fatty acids and amino acids as its main energy source [53]. Enzymes required for the *de novo *synthesis of inositol and mannose are important for amastigote growth [54]. In agreement with this, the myo-inositol-1-phosphate synthetase gene encoding a key enzyme in the first step of inositol synthesis was found upregulated in both *L. major *and *L. infantum *amastigotes (Table 2).

The absence of the glyoxylate pathway in *Leishmania*, which is required for the conversion of acetyl-CoA into sugars, indicates that amastigotes may be unable to utilize fatty acids as their major carbon source [19,55]. Amino acids are the second carbon source for *Leishmania *promastigotes but are also important in the amastigote stage [56-58]. The phagolysosomes of macrophages contain high levels of amino acids generated by proteolytic digestion of host phagosomal proteins and from exogenous proteins delivered into the phagolysosome via endocytosis [54,59]. Consistently, several lysosomal aminopeptidases and cathepsin-L like cysteine proteinases were upregulated in amastigotes (see Additional files 2 and 4). Also, several amino acid transporters and permeases important for the transport of amino acids from the phagosome into amastigotes were found upregulated in amastigotes (see Additional files 2 and 4 and [58]). *Leishmania *amastigotes can scavenge all their purine requirements, cations, vitamins, carbon sources and several essential amino acids from the macrophage phagolysosome via plasma membrane transporters [56]. Consistent with this, intracellular amastigotes of both *L. major *and *L. infantum *overexpress a relatively larger number of membrane transporters than promastigotes (see Additional files 2 and 4).

Important differences were observed in the expression of genes involved in cellular organization, biogenesis and cell motility between promastigote and amastigote stages of both *Leishmania *species. The motile flagellated promastigotes as opposed to the aflagellated amastigotes overexpress dyneins corresponding to large minus-end-directed microtubule motors providing the force for flagellar movement [30,31], microtubules and a variety of microtubule-associated proteins [60], kinesins and the trypanosomatid-specific PFR genes (Table 2, Additional files 1 and 3). The genes coding for microtubule-associated proteins were found upregulated in *L. major *but surprisingly not in *L. infantum *promastigotes (Table 4). The genes coding for calpains, calcium-dependent cysteine proteinases participating in a variety of cellular processes, including cytoskeletal/membrane attachments and signal transduction pathways [61], were upregulated in *L. major *and *L. infantum *promastigotes (Table 2). In contrast, intracellular amastigotes upregulated the expression of the lysosomal cathepsin-L like cysteine proteinases or aminopeptidases (see Additional files 2 and 4).

### Species-specific differential gene expression

Comparative analyses of the complete genomes of *L. major*, *L. infantum *and *L. braziliensis *causing distinct human diseases revealed marked sequence conservation and synteny (e.g. more than 99% of genes between the three genomes were syntenic and conservation within coding sequences was 82–94%) [20]. Despite the 20–100 million years of divergence within the *Leishmania *genus and the broad difference in disease pathologies, only 78 genes were found to be species-specific; 5 *L. major*-specific, 26 *L. infantum*-specific and 47 *L. braziliensis*-specific [20]. Remarkably, our studies revealed that some of these unique species-specific genes, all encoding hypothetical proteins, were also developmentally regulated. For example, LinJ22_V3.0670 only present in *L. infantum*, or LinJ34_V3.3430, LinJ15_V3.0620, LinJ16_V3.1460, and LinJ31_V3.1180 present in *L. infantum *but pseudogenes in *L. major *[20] were expressed preferentially in the amastigote stage (see Additional file 2). The *L. major *unique genes LmjF04.1020 and LmjF32.2470 were differentially expressed in promastigotes and amastigotes, respectively. The *L. major *promastigote-regulated genes LmjF10.0185 and LmjF27.0870 are pseudogenes in *L. infantum *(see Additional file 3). Few of these genes are also present in *L. braziliensis *although their developmental regulation, if any, is not known. Interestingly, the *L. donovani *A2 genes (pseudogenes in *L. major*), the only genes implicated so far in disease tropism [62], are specifically expressed in amastigotes [11]. These data suggest that both parasite genome differences and differential expression of species-specific genes may contribute to species-specific tropism.

The comparative transcriptomic analyses between *L. major *and *L. infantum *showed that only 10–12% of the differentially expressed genes in either life stage were common to both species. Commonly upregulated genes may fulfill essential features of the parasite such as metabolism, motility, infectivity and interaction with the host. Consistently, several metabolic genes, the amastigote-specific amastin surface proteins, which may act as receptors or transporters/channels [15] and the promastigote-upregulated dyneins, paraflagellar rod components and calpain-like cysteine peptidases were differentially upregulated in both species. The family of receptor adenylase cyclases (RAC) that play a role in signal transduction was differentially upregulated both in *L. major *and *L. infantum *promastigotes (Table 2). This is consistent with the report that rac-A and rac-B genes were expressed preferentially in the insect stage of *L. donovani *[63]. However, the majority of the differentially expressed genes in either life stage were specific to each *Leishmania *species. Species-specific differential gene expression may be attributed to a dynamic mode of regulation, which is suitable to species-specific adaptations to different insect vectors and life-cycle features, different target tissues, and distinct disease pathogenesis. Important differences in the stage-regulated gene expression of RNA-binding proteins, amino acid transporters, proteolytic enzymes, and protein kinases were observed between *L. major *and *L. infantum*. For example, *L. major *amastigotes upregulate the expression of important virulence genes encoding the lysosomal cathepsin-L like cysteine proteinases of the papain superfamily [64] whereas *L. infantum *amastigotes overexpress genes encoding aminopeptidases, which catalyze the removal of N-terminal amino acid residues from peptides and proteins, as well as the subtilisin-like serine peptidase and the hslvu complex proteolytic subunit (see Additional files 2 and 4). Members of the surface antigen proteins 2 (or GP46) are upregulated in *L. major *promastigotes but not in *L. infantum *promastigotes (Tables 3, 4 and Additional files 1 and 4).

It is likely that lesion-derived amastigotes and amastigotes isolated from *in vitro*-infected macrophages display some differences in the expression of genes, especially those that can be modulated by the immune status of the host and/or gene products that contribute together with host factors to the establishment of the disease. However, the majority of modulated genes whose functions are important or essential for the parasite's differentiation into its amastigote form and for the survival of amastigotes within the phagolysosome of macrophages should in principle be modulated similarly in lesion-derived or in vitro-infected macrophages given that amastigotes isolated from THP1 at 4–5 days following infection are fully differentiated and divide within the phagolysosome. The substantial differences that we observed in stage-regulated gene expression not only for amastigotes but also for promastigotes, which were cultured under the same conditions, between *L. infantum *and *L. major *may be attributed for the most part to species-specific factors. In line with this possibility are recent observations from our laboratory indicating that gene orthologues between *L. major *and *L. infantum *can be regulated in a different manner implying either distinct 3'UTR elements or possibly regulatory factors that are only expressed in one of the species (Müller *et al*., unpublished). In further agreement with our findings, is a recent interspecies microarray analysis comparing lesion-derived amastigotes of *L. major *and *L. mexicana, *two species causing cutaneous leishmaniasis, which demonstrated substantial differences in RNA expression profiles. Indeed, only 15% of the differentially expressed genes in promastigotes were in common between *L. major *and *L. mexicana *and there were no amastigote-upregulated genes shared by the two species [65].

We showed here, and others have reported previously [25-29], that a small percentage of genes (7–9%) are regulated at the RNA level throughout the life cycle stages of *Leishmania*. This percentage of modulated genes is not negligible if one considers that regulation of gene expression in *Leishmania *occurs at different levels. Several proteomics studies reported a higher amount of genes (12–18%) being regulated in a stage-specific manner at the translational and posttranslational levels [65-67]. It has also been reported that a modest correlation exists between mRNA and protein levels [66]. This modest correlation could be partly explained by the presence of multiple elements within the 3'UTR of *Leishmania *transcripts, which often have distinct functions on either mRNA stability or translation [46,48,68].

### Do stage-regulated transcripts share common regulatory motifs in their 3'UTR?

It is now well established that stage-specific regulation of gene expression in *Leishmania *is predominantly mediated by elements in the 3'UTR of mRNAs (reviewed in [22,23]). In few cases, specific regions within 3'UTRs have been delineated as *cis*-elements but their mechanism of action remains for the most part unknown. We recently reported the presence of two classes of distinct short interspersed degenerate retroposons, SIDER1 and SIDER2 that are widely distributed in the 3'UTR of a large number of *Leishmania *mRNAs (Table 6) [46]. We showed that SIDER1 and SIDER2 have distinct regulatory functions involving either mRNA translational control or RNA degradation, respectively [46-48]. SIDERs were the first conserved regulatory elements found in numerous *Leishmania *transcripts. However, the distribution of SIDERs among differentially expressed transcripts was not significantly higher from their global distribution among *Leishmania *transcripts (~25%) (Table 6) [46] (Smith, M. *et al*., unpublished). SIDER1-mediated regulation occurs at the level of translation and does not necessarily affect mRNA abundance [48]. In the few cases studied, SIDER2 seems to be involved in the degradation of generally short-lived mRNAs. Rapid mRNA turnover may permit the parasite to adapt to the continuously changing physiological needs within its invertebrate and mammalian host, and these temporal changes may not be detectable under our experimental conditions. Hence, SIDERs might participate in more dynamic regulatory processes throughout the *Leishmania *life cycle in response to specific environmental stimuli, yet to be analyzed. It is likely that additional 3'UTR elements should regulate stability of the differentially expressed transcripts in *Leishmania*. For example, we have recently identified a U-rich element in the amastin 3'UTR that contributes to the amastin mRNA degradation specifically in promastigotes [68].

## Conclusion

In conclusion, whole-genome analyses of inter-stage and inter-species RNA expression profiles between *L. major *and *L. infantum *promastigote and amastigote developmental forms highlighted that only a small percentage of *Leishmania *transcripts are modulated during development and most importantly that *Leishmania *species causing distinct pathologies express a diverse set of differentially expressed transcripts. These important differences in stage-regulated gene expression between species may be necessary to allow species-specific adaptations to different insect vectors and life-cycle features and could contribute to disease tropism. It is possible that in order to diversify and/or to enlarge their expression profile patterns in response to continuously changing environments throughout development, *Leishmania *species have adopted a dynamic, multilayered and complex mode of regulating gene expression. The results presented in this study favor this possibility.

## Methods

### *Leishmania *strains, cell culture and differentiation

The *L. infantum *MHOM/MA/67/ITMAP-263 and *L. major *LV39 MRHO/SU/59/P strains used in this study have been previously described [69,70]. *L. infantum *and *L. major *promastigotes were cultured at pH 7.0 and 25°C in SDM-79 medium [71] supplemented with 10% heat-inactivated fetal calf serum (Multicell, Wisent Inc) and 5 μg/ml hemin. *L. infantum *amastigotes were cultured in a human leukemia monocyte cell line (THP-1 cells) as described previously [72]. THP-1 cells in the log phase of growth were differentiated by incubation for 2 days in medium containing 20 ng of phorbol myristate acetate/ml (Sigma). THP-1 cells were then washed with pre-warmed medium and subsequently infected with stationary-phase *L. infantum *promastigotes in flat bottom tissue culture flasks (75 cm^2^) at a parasite/macrophage ratio of 15:1. After two hours, non-internalized parasites were removed by several washes, and infected macrophages were incubated for 4 days. *L. major *amastigotes were also cultured in THP-1 using the same protocol. *L. major *lesion amastigotes were obtained from the infected footpad of BALB/c mice 6–8 weeks post-infection as previously described [70].

### RNA extraction and cDNA sample preparation

Infected macrophages were incubated with cold HEPES-NaCl-0.0125% SDS for few minutes and neutralized with cold HEPES-NaCl. After centrifugation at 3000 rpm, macrophages were resuspended in cold HEPES-NaCl and, on ice passed 10 times in syringes equipped with a 27G1/2 needle to obtain pure amastigotes free of macrophage material. Amastigotes were washed once in cold HEPES-NaCl and total RNA was prepared using RNeasy Plus (Qiagen) as instructed by the manufacturer. *L. major *lesion amastigotes were extracted from macrophages using the same procedure as above. Total RNA from promastigotes grown to mid log phase was prepared using RNeasy Plus directly. RNA was treated with Turbo DNAse-free (Ambion). The quality and quantity of RNA were assessed using RNA 6000 Nano Assay Chips (Agilent Technologies). The presence of three distinct ribosomal peaks (18S, 24Sα and 24Sβ) confirmed successful RNA extraction and no detectable macrophage RNA in the *L. infantum *and *L. major *amastigote RNA preparations. Complementary DNA (cDNA) was generated from 5 μg of total RNA using a random primer hexamer (GE Healthcare) and aminoallyl-dUTP mix (Sigma) following the protocol for Superscript III (Invitrogen). After 5 hours of incubation at 50°C, the RNA was hydrolyzed with NaOH and EDTA for 15 min at 65°C, then pH was neutralized with HCl. cDNA was purified from unincorporated aminoallyl-dUTP with MinElute PCR Purification columns (Qiagen), phosphate wash buffer (5 mM KPO_4 _pH 8.0, 80% EtOH) and phosphate elution buffer (4 mM KPO_4 _pH 8.5).

### *L. infantum *and *L. major *DNA oligonucleotide full genome microarray design

The recent completion of the sequence of the *L. major *[19] and *L. infantum *genomes [20] allowed the generation of multispecies high-density oligonucleotide microarrays. The genome of both species was first compared using BLAST and genes on the same chromosome that had more than 95% homology were grouped together. 70-mer oligonucleotides were designed for each open reading frame of *L. infantum *and *L. major *with consistent thermodynamic properties using automated bioinformatic procedures. Probes were initially designed for *L. infantum *with the added requirement that the region targeted by the probe had optimal homology between both species. For common probes, up to two mismatches were tolerated. In the case that more than 2 mismatches were present in a given gene between *L. infantum *and *L. major*, a new probe was designed specifically for *L. major *(956 probes). The microarray includes in total 8,978 70-mer probes that recognize with no mismatches all *L. infantum *genes (8,184, GeneDB version 3.0) and also all *L. major *genes (8,370 genes, GeneDB version 5.1) with the majority of the probes sharing no mismatches and a small percentage of the probes having at most two mismatches (3,580 genome probes are 100% identical between *L. infantum *and *L. major *and 3,410 probes have 1–2 mismatches). Also, 622 control probes were included in the microarray for assessing synthesis variability, location of the probe within a given open reading frame and number of mismatches. The probes were synthesised in 384-well plates by Invitrogen (Burlington, Canada). The microarrays were printed on SuperChip (Erie Scientific) using a BioRobotics MicroGrid (Genomic solutions Inc, Ann Arbor, MI). Each probe was printed in duplicate. To assess the ubiquity and specificity of the oligonucleotide probes, the DNA microarray was hybridized in triplicate with genomic DNA isolated from either *L. infantum *or *L. major*. Following scanning, both genomic DNA preparations hybridized more than 99% of the oligonucleotides on the array (data not shown). All microarray data will be freely available on the Geo NCBI database in the MIAME format [73]. The series accession number for our manuscript is GSE10407.

### Preparation of labeled cDNA and microarray hybridization

Probes for DNA microarray hybridizations were prepared with 10 μg of total RNA for each condition. Purified cDNA from either promastigotes or intracellular amastigotes was dried in speed-vac and resuspended in 0.2 M sodium bicarbonate buffer pH 9.3. Alexa 555 and Alexa 647 dye (Invitrogen) was dissolved in DMSO and incubated in dark with either cDNA for 2 h at room temperature. After coupling, sodium acetate was added and cDNA was purified with MinElute PCR Purification columns (Qiagen) and washed with phosphate washing buffer and phosphate elution buffer. The array was pre-hybridized with 5× complete Denhardt, 30% deionized formamide, 6× SSPE, 0.5% SDS and 0.1 mg/ml ssDNA for 1 h at 42°C and then washed twice for 5 min. at 42°C with 2× SSC, 0.1% SDS, 3 min. at 25°C with 1× SSC, 3 min. at 25°C with 0.2 × SSC and 3 min at 25°C with 0.05 × SSC. Hybridizations were carried out overnight at 42°C (2.5× modified Denhardt, 30% deionized formamide, 6× SSPE, 0.5% SDS, 0.1 mg/ml ssDNA and 0.75 mg/ml yeast tRNA). Multiple biological replicates (4 for *L. major *and 6 for *L. infantum*) of all hybridizations were performed to account for sample heterogeneity, variation between slides and variations due to hybridization. To prevent bias by preferential label incorporation into particular sequences, Alexa 555 and Alexa 647 dyes were swapped between the two RNA preparations. Hybridized slides were washed using the pre-hybridization buffer described above.

### Fluorescence detection, data processing and statistical analysis

The fluorescence signal intensities of six slides hybridized with *L. infantum *RNA isolated from promastigotes or amastigotes and four slides hybridized with the *L. major *RNA (promastigote and amastigote) were measured using the Perkin Elmer ScanArray Express Scanner. GenePix Pro 6.0 image analysis software (Axon Instruments, Union City, California, United States) was employed to measure the fluorescence signal intensities of the array features and local background. Raw data from GenePix were imported in R 2.2.1 for normalization and statistical analyses using the LIMMA (version 2.7.3) package [74-76]. Before processing, probes were flagged according to their hybridization quality on the *Leishmania *species [43]. Weights were assigned to each array in order to give less weight to arrays of lesser quality [77]. Data were corrected using background subtraction based on convolution of normal and exponential distributions [78]. Intra-array normalization was carried out using the "print-tip loess" method and inter-array normalization was done using the "quantiles of A test" for each array [79]. Statistical analysis was done using linear model fitting and standard errors were moderated using a simple empirical Bayes [74]. Multiple testing corrections were done using the False Discovery Rate (FDR) method with a threshold *p*-value of 0.05. Only genes statistically significant with an absolute log2 ratio greater than 0.75 were considered as differentially expressed. Further species comparison was performed only on probes that had less than two mismatches when hybridized to either *Leishmania *species. Thus, 6990 probes could be directly compared between the two species. Gene ontology annotation was analyzed using the AmiGO website [80].

### Quantitative Real-Time RT-PCR

Quantitative real-time PCR was carried out on a selected number of genes for validating the microarray experiments (see Table 5 and Additional file 5). Three independent RNA preparations (same used for the microarray experiments) were conducted for each condition. First-strand cDNA was synthesized from 2 μg of total RNA using the Superscript III RNase H Reverse Transcriptase enzyme and random hexamers (GE Healthcare) according to manufacturer's instructions. The resulting cDNA samples were stored at -20°C until use. The primers were designed using Primer Express 2.0 (Applied Biosystems) and their sequence data are the following: × gene: 5-Ztail-N-3; Y gene: 5-Ztail-N-3 (see Additional file 6). Equal amounts of cDNA were run in triplicate and amplified in a 15 μl reaction containing 7.5 μl of 2× universal PCR master mix (Applied Biosystems, Foster City, CA), 10 nM of Z-tailed forward primer, 100 nM of reverse primer, 250 nM of Amplifluor Uniprimer probe (Chemicon, Temecula, California), and 1 μl of cDNA target. Reactions were performed at the Gene Quantification Core Laboratory of the Centre de Génomique de Québec using the Applied Biosystems Prism 7900 Sequence Detector [81]. Three technical and two biological replicates of each reaction were performed, amplification efficiencies were validated and gene expression levels were normalized to constitutively expressed mRNAs encoding the ubiquitin hydrolase gene (LinJ29_V3.2410/LmjF29.2300), 60S ribosomal protein (LinJ18_V3.0630/LmjF18.0620) and a hypothetical protein (LinJ31_V3.0190/LmjF31.0180). Quantification of the relative changes in target gene expression was calculated according to a standard curve.

## Authors' contributions

AR performed the microarray experiments, did the mice infections, prepared all Figures and Tables, and participated in the analysis of the data and the writing of the manuscript. FR performed most of the data analysis and participated in the design of the oligonucleotide probes. J-MU established the microarray protocol and helped in the design of the microarray experiments. NM did the macrophage infections. SB helped in the design of the oligonucleotide probes for the construction of the *Leishmania *full-genome array. MS did the bioinformatics studies on SIDER's distribution among the developmentally regulated transcripts. PR did all the initial bioinformatics work comparing the two *Leishmania *genomes to design the appropriate oligonucleotide probes for the array. JC and MO participated in the design of the study and reviewed the manuscript. BP participated in the design of the study, coordinated many aspects of the study and wrote the manuscript. All authors have read and approved the final manuscript.

## Supplementary Material

Additional file 1 **Genes differentially expressed in *Leishmania infantum *promastigotes**. This Table lists all the *Leishmania infantum *genes that are differentially expressed in promastigotes as determined by DNA microarray studies. Click here for file

Additional file 2 **Genes differentially expressed in *Leishmania infantum *intracellular amastigotes**. This Table lists all the *Leishmania infantum *genes that are differentially expressed in intracellular amastigotes as determined by DNA microarray studies. Click here for file

Additional file 3 **Genes differentially expressed in *Leishmania major *promastigotes**. This Table lists all the *Leishmania major *genes that are differentially expressed in promastigotes as determined by DNA microarray studies. Click here for file

Additional file 4 **Genes differentially expressed in *Leishmania major *intracellular amastigotes**. This Table lists all the *Leishmania major *genes that are differentially expressed in lesion-derived amastigotes as determined by DNA microarray studies. Click here for file

Additional file 5 **Comparison of expression levels obtained by quantitative real-time PCR between different *Leishmania *species or experimental models of infection**. qRT-PCR analysis was performed on selected differentially expressed genes as determined by microarray experiments. The same RNA used for the microarray analysis was also used for qRT-PCR. (A) Expression values of selected genes in *L. major *amastigotes isolated either from mouse lesions or from THP1-infected macrophages as determined by qRT-PCR. (B) The qRT-PCR gene expression values of selected genes differentially expressed in *L. infantum *and/or *L. major *amastigotes isolated from THP1-infected macrophages. The data are presented as an amastigote to promastigote (Ama/Pro) ratio. Two biological replicates and three technical replicates were included. Error bars denote standard deviations. Values without error bars represent standard deviations lower than 0.05. Click here for file

Additional file 6 **Primers used for quantitative real-time PCR expression analysis**. Table lists the sequences of the primers used for quantitative real-time PCR expression analysis to validate DNA microarray studies. Click here for file
