# Supplementary material for: Genome-wide gene expression profiling analysis of Leishmania major and Leishmania infantum developmental stages reveals substantial differences between the two species
Source: BMC Genomics. 2008 May 29;9:255. doi: 10.1186/1471-2164-9-255 (PMC2453527; doi:10.1186/1471-2164-9-255)
Supplement: Additional file 1 — Genes differentially expressed in Leishmania infantum promastigotes. This Table lists all the Leishmania infantum genes that are differentially expressed in promastigotes as determined by DNA microarray studies. [file 1471-2164-9-255-S1.doc]

**Table S1.** Genes differentially expressed in *Leishmania infantum* promastigotes.

^a^ Gene functions are based on Gene Ontology (GO) annotation. The main categories are shown here. In some cases, some categories include sub-categories like the carbohydrate metabolic process includes glycolysis, galactose metabolism and inositol metabolism.

^b^ A single probe within the open reading frame was used to recognize the multicopy genes.
