# Supplementary material for: Genome-wide gene expression profiling analysis of Leishmania major and Leishmania infantum developmental stages reveals substantial differences between the two species
Source: BMC Genomics. 2008 May 29;9:255. doi: 10.1186/1471-2164-9-255 (PMC2453527; doi:10.1186/1471-2164-9-255)
Supplement: Additional file 2 — Genes differentially expressed in Leishmania infantum intracellular amastigotes. This Table lists all the Leishmania infantum genes that are differentially expressed in intracellular amastigotes as determined by DNA microarray studies. [file 1471-2164-9-255-S2.doc]

**Table S2.** Genes differentially expressed in *Leishmania infantum* intracellular amastigotes.

^a^ As in Additional file 1.

^b^ Not all the phosphoglycan beta 1,3 galactosyltransferase memebers are differentially expressed in the amastigote stage. Our probe was designed to recognize LinJ14_V3.1500 but it can also recognize (only few mismatches) 5 other copies (LinJ02_V3.0140, LinJ02_V3.0150, LinJ02_V3.0160, LinJ02_V3.0170 and LinJ02_V3.0180). A specific probe showed that LinJ02_V3.0140 is differentially expressed in the promastigote stage.

^c^ A single probe within the open reading frame was used to recognize the multicopy genes.

^d^ A specific probe was designed for each amastin gene but due to high sequence identity in some cases, this probe could also recognize other family members. A probe designed for LinJ08_V3.0680 can recognize LinJ08_V3.0690, LinJ08_V3.0720, LinJ08_V3.0780, LinJ08_V3.1320, LinJ31_V3.0460, LinJ34_V3.1010, LinJ34_V3.1030, LinJ34_V3.1670, LinJ34_V3.2650, LinJ34_V3.2660 and LinJ34_V3.4340. A probe designed for LinJ08_V3.0690 and LinJ08_V3.0720 can recognize LinJ08_V3.0680, LinJ08_V3.1320 and LinJ31_V3.0460. A probe designed for LinJ08_V3.0700, LinJ08.0710 and LinJ08_V3.1330 can recognize LinJ08_V3.0760, LinJ08_V3.0790, LinJ34_V3.1010, LinJ34_V3.1030, LinJ34_V3.2650, LinJ34_V3.2660, LinJ34_V3.4340 and LinJ34_V3.4350. A probe designed for LinJ08_V3.0760 can recognize LinJ08_V3.0780 and LinJ08_V3.0790. A probe recognizes both copies LinJ29_V3.1450 and LinJ29_V3.3000. A probe designed for LinJ34_V3.1010 and LinJ34_V3.1030 can recognize LinJ08_V3.0700, LinJ08_V3.0710, LinJ08_V3.0760, LinJ08_V3.0780, LinJ08_V3.0790, LinJ08_V3.1330, LinJ34_V3.1150, LinJ34_V3.1670, LinJ34_V3.1700, LinJ34_V3.1720, LinJ34_V3.2650, LinJ34_V3.2660, LinJ34_V3.4340, LinJ34_V3.4350 and LinJ34_V3.4370. A probe designed for LinJ34_V3.1020 can also recognize LinJ34_V3.1010, LinJ34_V3.1030, LinJ34_V3.1150 and LinJ34_V3. 4370. A probe designed for LinJ34_V3.1150 can recognize LinJ34_V3.1010, LinJ34_V3.1030 and LinJ34_V3.4370. A probe designed for LinJ34_V3.1680 can recognize LinJ34_V3.1690, LinJ34_V3.1700, LinJ34_V3.1710 and LinJ34_V3.1730. A probe designed for LinJ34_V3.1700 can recognize LinJ34_V3.1710. A probe designed for LinJ34_V3.1710 can recognize LinJ34_V3.1670, LinJ34_V3.1680, LinJ34_V3.1700 and LinJ34_V3.1730. A probe designed for LinJ34_V3.2660 can recognize LinJ29_V3.3030. A probe designed for LinJ34_V3.4340 can recognize LinJ08_V3.0680, LinJ08_V3.0690, LinJ08_V3.0720, LinJ08_V3.1320, LinJ34_V3.1010, LinJ34_V3.1030, LinJ34_V3.1150, LinJ34_V3.4350 and LinJ34_V3.4370. A probe designed for LinJ34_V3.4370 can recognize LinJ08_V3.0680, LinJ08_V3.0690, LinJ08_V3.0720, LinJ08_V3.0760, LinJ08_V3.0780, LinJ08_V3.1320, LinJ31_V3.0460, LinJ34_V3.1010, LinJ34_V3.1030, LinJ34_V3.1150, LinJ34_V3.1670, LinJ34_V3.1700, LinJ34_V3.4340 and LinJ34_V3.4350. A specific probe was designed for the following family members: LinJ08_V3.0780, LinJ08_V3.0790, LinJ29_V3.3010, LinJ31_V3.0460, LinJ34_V3.1670, LinJ34_V3.1720 and LinJ34_V3.1730.

^e^ *L. major* pseudogenes.

^f^ *L. infantum* unique genes.
