# Supplementary material for: Genome-wide gene expression profiling analysis of Leishmania major and Leishmania infantum developmental stages reveals substantial differences between the two species
Source: BMC Genomics. 2008 May 29;9:255. doi: 10.1186/1471-2164-9-255 (PMC2453527; doi:10.1186/1471-2164-9-255)
Supplement: Additional file 3 — Genes differentially expressed in Leishmania major promastigotes. This Table lists all the Leishmania major genes that are differentially expressed in promastigotes as determined by DNA microarray studies. [file 1471-2164-9-255-S3.doc]

Table S3. Genes differentially expressed in *Leishmania major* promastigotes.

^a^ As in Additional file 1.

^b^ Genes in bold correspond to differentially expressed genes in *L. major* Friedlin promastigotes identified previously by full-genome DNA oligonucleotide microarrays (Leifso *et al*. 2007).

^c^ As in Additional file 2.

^d^ Three probes recognize all the microtubule-associated proteins. The first probe was designed to recognize LmjF09.0150, LmjF09.0154, LmjF09.0158, LmjF09.0162, LmjF09.0166, LmjF09.0170 and LmjF09.0174. The second probe recognizes LmjF19.0820, LmjF19.0842, LmjF19.0846, LmjF19.0850, LmjF19.0880 and LmjF19.0910 can recognize LmjF19.0860, LmjF19.0870 and LmjF19.0900. A third probe recognizes LmjF19.0860, LmjF19.0870 and LmjF19.0900.

^e^ A probe designed for LmjF12.0870 recognizes LmjF12.0730, LmjF12.0755, LmjF12.0760, LmjF12.0765, LmjF12.0780, LmjF12.0810, LmjF12.0830, LmjF12.0850, LmjF12.0890, LmjF12.0940, LmjF12.0960, LmjF12.0980, LmjF12.0990, LmjF12.1000 and LmjF12.1070. Another probe recognizes LmjF12.0850 and LmjF12.1070. A probe designed for LmjF12.0740 can recognize LmjF12.0730, LmjF12.0765, LmjF12.0860, LmjF12.0870, LmjF12.0890, LmjF12.0910, LmjF12.0920, LmjF12.1020, LmjF12.1040, LmjF12.1060, LmjF12.1070 and LmjF12.1090. A probe designed for LmjF12.0760 can recognize LmjF12.0730, LmjF12.0740, LmjF12.0755, LmjF12.0780, LmjF12.0810, LmjF12.0830, LmjF12.0860, LmjF12.0890, LmjF12.0910, LmjF12.0920, LmjF12.0940, LmjF12.0960, LmjF12.0980, LmjF12.0990, LmjF12.1000, LmjF12.1020, LmjF12.1040, LmjF12.1060 and LmjF12.1090. A probe designed for LmjF12.0765 can recognize LmjF12.0740, LmjF12.0860, LmjF12.0870, LmjF12.0890, LmjF12.0910, LmjF12.0920, LmjF12.0990, LmjF12.1020, LmjF12.1040, LmjF12.1060 and LmjF12.1090. A probe designed for LmjF12.0830 can recognize LmjF12.0740, LmjF12.0765, LmjF08.0850, LmjF12.0960, LmjF12.1005 and LmjF12.1070. A probe designed for LmjF12.0860 can recognize LmjF12.0730, LmjF12.0740, LmjF12.0755, LmjF12.0760, LmjF12.0830, LmjF12.0910, LmjF12.0920, LmjF12.0960, LmjF12.0990, LmjF12.1020, LmjF12.1040 and LmjF12.1060. A probe designed for LmjF12.1020 can recognize LmjF12.0730, LmjF12.0740, LmjF12.0755, LmjF12.0830, LmjF12.0860, LmjF12.0960, LmjF12.0990, LmjF12.1040 and LmjF12.1060. A specific probe was designed for LmjF12.0730 and LmjF12.1090.

^f^ *L. major* unique genes.

^g^ *L. infantum* pseudogenes.
