# Supplementary material for: Genome-wide gene expression profiling analysis of Leishmania major and Leishmania infantum developmental stages reveals substantial differences between the two species
Source: BMC Genomics. 2008 May 29;9:255. doi: 10.1186/1471-2164-9-255 (PMC2453527; doi:10.1186/1471-2164-9-255)
Supplement: Additional file 4 — Genes differentially expressed in Leishmania major intracellular amastigotes. This Table lists all the Leishmania major genes that are differentially expressed in lesion-derived amastigotes as determined by DNA microarray studies. [file 1471-2164-9-255-S4.doc]

**Table S4.** Genes differentially expressed in *Leishmania major* lesion-derived amastigotes.

^a^ As in Additional file 1.

^b^ Genes in bold were previously identified as differentially regulated in *L. major* Friedlin lesion-derived amastigotes by full-genome DNA oligonucleotide microarrays (Leifso *et al*. 2007).

^c^ Not all phosphoglycan beta 1,3 galactosyltransferase genes are differentially expressed in the amastigote stage. A specific probe recognizing LmjF02.0160 showed that this gene is differentially expressed in the promastigote stage. A probe designed for LmjF02.0010 can also recognize 10 other copies (LmjF02.0160, LmjF02.0170, LmjF02.0200, LmjF02.0210, LmjF07.1170, LmjF21.0010, LmjF25.2460, LmjF31.3190, LmjF35.0010, LmjF36.0010). A probe designed for LmjF14.1400 can also recognized 4 other copies (LmjF02.0160, LmjF02.0170, LmjF02.0200, LmjF02.0210). A specific probe showed that LmjF31.3190 and LmjF35.0010 are differentially expressed in the amastigote stage.

^d^ A single probe within the open reading frame was used to recognize the multicopy genes.

^e^ A specific probe was designed for each amastin gene but due to high sequence identity in same cases this specific probe could recognize other family members. A probe designed for LmjF08.0670, LmjF08.0680, LmjF08.0690, LmjF08.0710, LmjF08.0730 and LmjF08.0750 can also recognize LmjF08.0700, LmjF08.0720, LmjF08.0740, LmjF08.0760 and LmjF08.0770. A probe designed for LmjF08.0700, LmjF08.0720, LmjF08.0740, LmjF08.0760 and LmjF08.0770 can also recognize LmjF08.0670, LmjF08.0680, LmjF08.0690, LmjF08.0710, LmjF08.0730 and LmjF08.0750. A probe designed for LmjF08.0800 and LmjF08.0840 can recognize LmjF08.0820. A probe designed for LmjF34.1580, LmjF34.1620, LmjF34.1660, LmjF34.1680, LmjF34.1880 and LmjF34.1960 can recognize LmjF34.1560, LmjF34.1600, LmjF34.1640, LmjF34.1700, LmjF34.1720, LmjF34.1740, LmjF34.1760, LmjF34.1780, LmjF34.1800, LmjF34.1820, LmjF34.1860, LmjF34.1900, LmjF34.1940, LmjF34.1980 and LmjF36.1270. A probe designed for LmjF34.1560, LmjF34.1740 and LmjF34.1900 can recognize LmjF34.1580, LmjF34.1600, LmjF34.1640, LmjF34.1660, LmjF34.1700, LmjF34.1720, LmjF34.1760, LmjF34.1780, LmjF34.1800, LmjF34.1820, LmjF34.1860, LmjF34.1920, LmjF34.1940, LmjF34.1980 and LmjF36.1270. A probe designed for LmjF34.1640 can recognize LmjF36.1270. A probe designed for LmjF34.1700, LmjF34.1760, LmjF34.1780, LmjF34.1800, LmjF34.1820, LmjF34.1860, LmjF34.1940 and LmjF34.1980 can recognize LmjF34.0500, LmjF34.0960, LmjF34.1080, LmjF34.1560, LmjF34.1580, LmjF34.1600, LmjF34.1620, LmjF34.1640, LmjF34.1660, LmjF34.1680, LmjF34.1720, LmjF34.1740, LmjF34.1880, LmjF34.1900, LmjF34.1920, LmjF34.1960 and LmjF36.1270. A probe designed for LmjF34.1720 can recognize LmjF34.1560, LmjF34.1580, LmjF34.1600, LmjF34.1620, LmjF3.1640, LmjF34.1660, LmjF34.1680, LmjF34.1700, LmjF34.1740, LmjF34.1760, LmjF34.1780, LmjF34.1800, LmjF34.1820, LmjF34.1860, LmjF34.1880, LmjF34.1900, LmjF34.1920, LmjF34.1940, LmjF34.1960, LmjF34.1980 and LmjF36.1270. A probe designed for LmjF36.1270 can recognize LmjF34.1640 and LmjF34.1920. A specific probe was designed for LmjF08.0810, LmjF08.0820, LmjF08.0850, LmjF24.1270, LmjF31.0450, LmjF34.0500, LmjF34.1080, LmjF34.1840 and LmjF34.1920.

^f^ *L. major* unique genes.
