# Supplementary figures and images for: Genome-wide gene expression profiling analysis of Leishmania major and Leishmania infantum developmental stages reveals substantial differences between the two species
Source: BMC Genomics. 2008 May 29;9:255. doi: 10.1186/1471-2164-9-255 (PMC2453527; doi:10.1186/1471-2164-9-255)

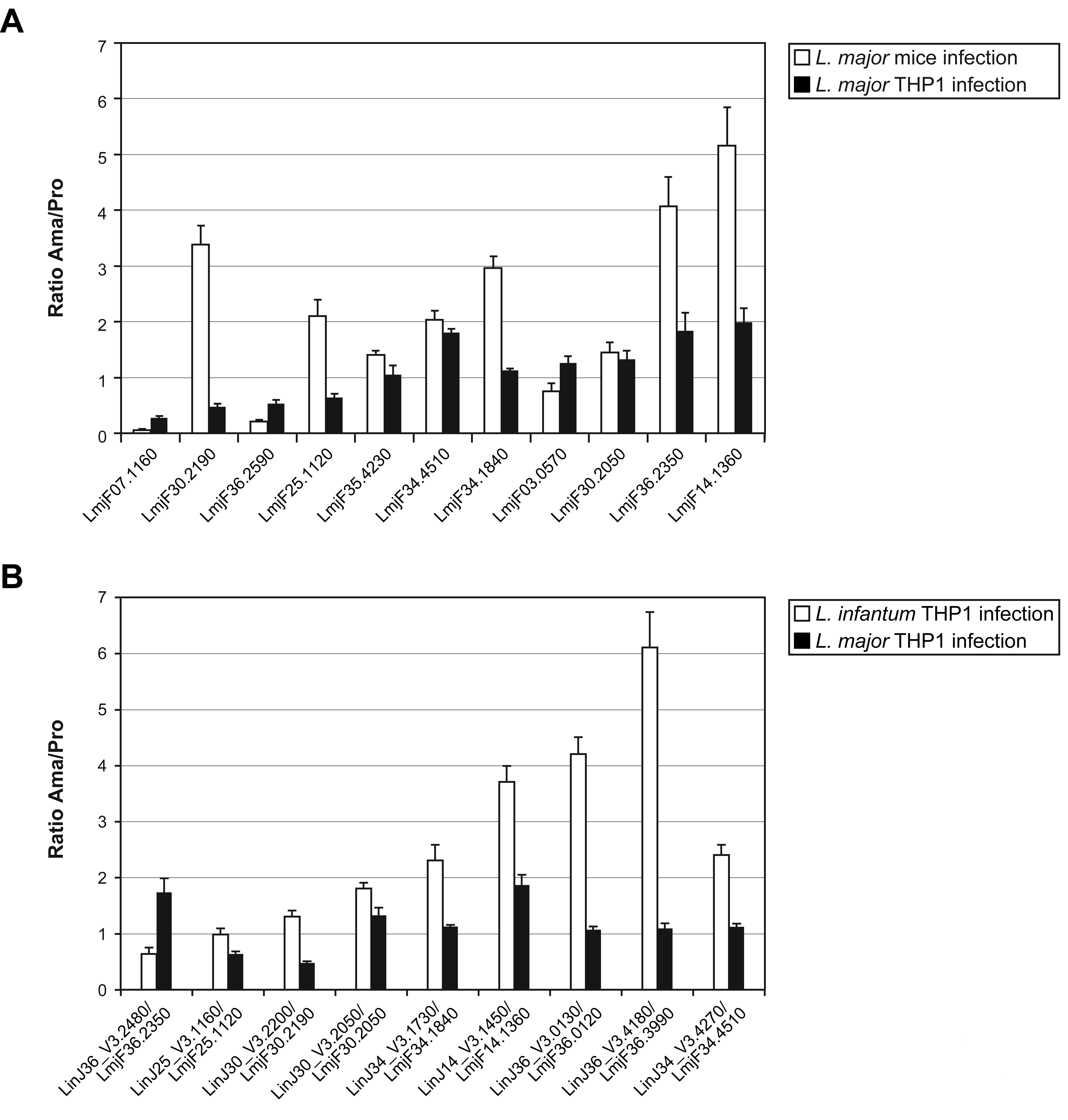

Supplement: Additional file 5 — Comparison of expression levels obtained by quantitative real-time PCR between different Leishmania species or experimental models of infection. qRT-PCR analysis was performed on selected differentially expressed genes as determined by microarray experiments. The same RNA used for the microarray analysis was also used for qRT-PCR. (A) Expression values of selected genes in L. major amastigotes isolated either from mouse lesions or from THP1-infected macrophages as determined by qRT-PCR. (B) The qRT-PCR gene expression values of selected genes differentially expressed in L. infantum and/or L. major amastigotes isolated from THP1-infected macrophages. The data are presented as an amastigote to promastigote (Ama/Pro) ratio. Two biological replicates and three technical replicates were included. Error bars denote standard deviations. Values without error bars represent standard deviations lower than 0.05. [file 1471-2164-9-255-S5.png]
