# Supplementary material for: Genome-wide gene expression profiling analysis of Leishmania major and Leishmania infantum developmental stages reveals substantial differences between the two species
Source: BMC Genomics. 2008 May 29;9:255. doi: 10.1186/1471-2164-9-255 (PMC2453527; doi:10.1186/1471-2164-9-255)
Supplement: Additional file 6 — Primers used for quantitative real-time PCR expression analysis. Table lists the sequences of the primers used for quantitative real-time PCR expression analysis to validate DNA microarray studies. [file 1471-2164-9-255-S6.doc]

**Table S5**: Primers used for quantitative real-time PCR expression analysis.

^a^ The same primers were used to detect *L. infantum* and *L. major* transcripts.

^b^ The underscore part in the primer is the z-tail used for detection.
